# Nanomaterial Gas Sensors for Online Monitoring System of Fruit Jams

**DOI:** 10.3390/foods8120632

**Published:** 2019-12-02

**Authors:** Estefanía Núñez-Carmona, Marco Abbatangelo, Ivano Zottele, Pierpaolo Piccoli, Armando Tamanini, Elisabetta Comini, Giorgio Sberveglieri, Veronica Sberveglieri

**Affiliations:** 1 CNR-IBBR, Institute of Bioscience and Bioresources, via Madonna del Piano, 10, 50019 Sesto Fiorentino, FI, Italy; estefania.nunezcarmona@ibbr.cnr.it (E.N.-C.); veronica.sberveglieri@ibbr.cnr.it (V.S.); 2 Department of Information Engineering, University of Brescia, Brescia, via Branze, 38, 25123 Brescia, BS, Italy; elisabetta.comini@unibs.it; 3 Menz&Gasser S.p.A., Sede Legale Zona Industriale, 38050 Novaledo (TN), Italy; ivano.zottele@menz-gasser.it (I.Z.); pierpaolo.piccoli@menz-gasser.it (P.P.); armando.tamanini@menz-gasser.it (A.T.); 4 Nano Sensor Systems, NASYS Spin-Off University of Brescia, Brescia, via Camillo Brozzoni, 9, 25125 Brescia, BS, Italy; giorgio.sberveglieri@nasys.it

**Keywords:** volatile organic compounds (VOCs), gas chromatography–mass spectrometry (GC–MS), jams, chemical sensors, nanowire gas sensors, linear discriminant analysis (LDA)

## Abstract

Jams are appreciated worldwide and have become a growing market, due to the greater attention paid by consumers for healthy food. The selected products for this study represent a segment of the European market that addresses natural products without added sucrose or with a low content of natural sugars. This study aims to identify volatile organic compounds (VOCs) that characterize three flavors of fruit and five recipes using gas chromatography–mass spectrometry (GC–MS) and solid-phase micro-extraction (SPME) analysis. Furthermore, an innovative device, a small sensor system (S3), based on gas sensors with nanomaterials has been used; it may be particularly advantageous in the production line. Results obtained with linear discriminant analysis (LDA) show that S3 can distinguish among the different recipes thanks to the differences in the VOCs that are present in the specimens, as evidenced by the GC–MS analysis. Finally, this study highlights how the thermal processes for obtaining the jam do not alter the natural properties of the fruit.

## 1. Introduction

The term “marmalade” comes from the Portuguese “marmelo,” which means “quince tree”. This method to preserve fruits was already appreciated in the times of the ancient Greeks, who had discovered that the pulp of fruits, acrid and almost inedible, once cooked became very sweet with a strong scent of honey. Hence “quince” which in Greek means “apple of honey”.

The decree 20 February 2004 n°50 implementation of Directive 2001/113/EC concerning jams of the European community defines it as “the mixture brought to a suitable gelled consistency of sugars and pulp of one or more species of fruit and water. It is a particular type of food preserve. The fruit, according to the law, must be fresh, intact and healthy, at the right point of maturation, clean and blunt; the roots of the ginger, tomato, rhubarb (limited to the edible parts of the stems), carrots, cucumbers, pumpkins, melons, and watermelons are equated to the fruit” [1].

Fruit jams are a growing market. In the last year, the export value of the countries that market in this sector was approximately $3.2 billion. In this scenario, European countries have “the lion’s share,” with a turnover linked to exports of $2 billion—equal to 61.1% of the global total—followed by Asian producers (20.2%), those in Latin America (9.3%), North America (5.6%), Africa (2.7%) and Oceania (1.1%) [2].

In Europe, Italy is the second largest exporter of jams (8.4% of total exports worth $270.3 million market), preceded by France (12%) and followed by Turkey (8%) [2]. An explanation for this success is probably the desire of consumers to search for natural and simple products and those that, if possible, are also good for health. The growth in market value appears to be driven above all by the sector of jams without added sugar. Also, in the jam or marmalade market, as in general in many food markets, we are witnessing a shift towards increasing trade and purchase of “healthy” and less processed products, with clean ingredient lists and lower sugar content. The greater attention to diet products and increased awareness of the need to fight diseases such as diabetes with prevention are increasingly pushing the offer towards high-added-value light products.

Research on the volatile compounds of food aims to provide the characterization of aromatic profiles, thus allowing the identification of the most important compounds in defining the characteristics of the product [3,4]. The main components of food (proteins, amino acids, carbohydrates, lipids, and fatty acids) undergo degradation processes following conservation processes (e.g., seasoning) or due technological treatments (e.g., cooking), and thus arise a wide range of compounds such as hydrocarbons, esters, aldehydes, ketones, alcohols, nitrogen and sulfur compounds, which impart the characteristic aroma to the food [5,6,7].

The determination of volatile substances in food plays a role of considerable importance: these substances are in fact responsible for the aroma of the product, which can fall within the norms of acceptability and even be a peculiar characteristic of the product or anomalies due to the presence of substances which impart an unpleasant smell—the so-called “off-flavors” with chemical or microbiological origin [4]. It is therefore particularly important to identify and quantify the compounds normally present in the volatile fraction of food products in order to characterize the aromatic profile and to study the variations depending on, for example, the geographical origin, production technology, seasoning, ripening or interaction with the packaging material [8,9].

Volatile compounds can be extracted using different sampling techniques, both classic and innovative, such as dynamic headspace and solid-phase microextraction. In this study, both will be used in parallel. In fact, they will constitute the fundamental database that will pave the base for the algorithm design that will constitute the neural network capable of supporting the production process of jams.

Although very precise, the analysis methods currently used, are costly in terms of expensive instrumentation, at the time of analysis as well as to obtain a result, without neglecting the need to have prepared technical personnel [10,11,12,13,14,15]. The rapid methods currently available are instead still considered unsatisfactory, both due to the long analysis time and to the poor reliability and high costs. For many technologies currently in use, the high-speed response often recalls a lack of sensitivity or accuracy.

Currently, to the authors’ knowledge, just a few articles in the literature have dealt with the evolution of volatile compounds in food products of this type. For this reason, it is not only important to identify new rapid technologies and devices to support the production of high quality, but also to increase knowledge on their aromatic product to better understand the evolution that can occur during the cooking of the fruit used as raw material. As a result, interest in new technologies based on chemical sensor arrays [16] has grown in recent years. Ample interest has been demonstrated by the numerous scientific publications that are distributed both between classes of foods such as meats, vegetables, cereals, etc., but also between raw materials and finished and packaged products, following the entire production chain from the fields to the fork. Applications take into consideration geographical origins, production anomalies, supply chain checks or possible chemical and physical contamination of the matrix [17,18,19].

The aim of this study is to provide the characterization of the aromatic profile of jams, thus allowing the identification of compounds that most contribute to the identification of a particular product. In summary, this study will characterize the volatile compounds emitted by different jams using gas chromatography–mass spectrometry (GC–MS) with solid-phase micro-extraction (SPME) analysis. The jams used will be representative of both different flavors (the type of fruit used) and different recipes (types and percentage of ingredients). In addition, we will try to identify an innovative technique based on nanowire gas sensors, which can be an advantageous online decision-making aid to the business transformation process.

## 2. Materials and Methods

### 2.1. Experiment Design

**Figure 1:**
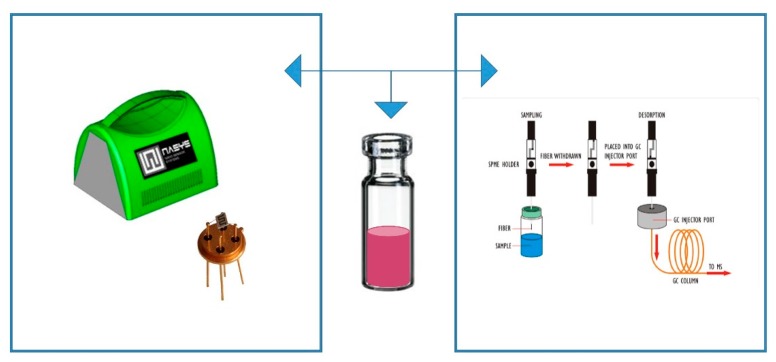
Illustration of online control of fruit jam by using a small sensor system (S3) device (**left**) and gas chromatography–mass spectrometry (GC–MS) and solid-phase micro-extraction (SPME) (**right**).

The research works were carried out following three steps. Firstly, the volatile organic compound (VOC) determination of fruit jams was carried out with GC–MS and SPME analysis (for details, see Section 2.2). Secondly, a small sensor system (S3) nanowire gas sensor device was developed and optimized (details see Section 2.3) in our lab with collaboration with the NANO SENSOR SYSTEMS Srl spin-off of the University of Brescia, Italy. The measures used in parallel, in this study, are shown in Figure 1.

In Table 1 flavors (strawberry, apricot, and cherry) with their recipes are shown. Samples were prepared under sterilized conditions. In total, 10 g of a sample was placed in a sterilized vial, sealed with septa made of polytetrafluoroethylene (PTFE)/silicone. Once closed, the samples were placed at room temperature for 1 hour before being analyzed in order to create a balanced headspace inside the vial. Vials, septa, syringes and all the material used for the preparation of the samples were sterilized in an autoclave at 121 °C for 15 min, in order to eliminate any kind of cross-microbiological contamination, which could alter the characteristic volatile organic compounds (VOCs) of the product, for the duration of the analysis. Samples used were treated and prepared for both techniques, exactly in the same way, to try to reduce the variables related to the preparation as much as possible. The operational conditions were interpreted in Section 2.3 and Section 2.4, respectively.

### 2.2. GC–MS SPME Detection

One hour after closure, vials were placed in the autosampler HT280T (HTA s.r.l., Brescia, Italy) to proceed with vial conditioning and volatile organic compound (VOC) extraction. Conditioning of the sample was performed as follows: filled vials where maintained for 15 min at 40 °C in order to equilibrate the headspace (HS) of the sample and to remove any variables. Afterward, VOC extraction was performed using solid-phase microextraction (SPME) analysis and the fiber used for the adsorption of volatiles was a divinylbenzene/carboxen/polydimethylsiloxane (DVB/CAR/PDMS) 50/30 µm (Supelco Co. Bellefonte, PA, USA) placed on the HT280T autosampler. The fiber was exposed to the vial HS in the HT280T oven thermostatically regulated at 40 °C for 30 min.

The GC instrument used in this work was a Shimadzu GC 2010 PLUS (Kyoto, KYT, Japan), equipped with a Shimadzu single quadrupole mass spectrometer (MS) MS-QP2010 Ultra (Kyoto, KYT, Japan). Fiber desorption took place in the GC–MS injector for 6 min at 250 °C. GC was operated in the direct mode throughout the run, while the separation was performed on a MEGA-WAX capillary column, 30 m × 0.25 mm × 0.25 μm, (Agilent Technologies, Santa Clara, CA, USA). Ultrapure helium (99.99%) was used as the carrier gas, at a constant flow rate of 1 mL/min. The GC oven temperature programming was applied as follows: at the beginning, the chromatographic column was held at 40 °C for 2 min and, subsequently, the temperature was raised from 40 to 70 °C at 5 °C/min, and held for 1 min. Next, the temperature was raised from 90 to 240 °C, with a rate of 10 °C/min; finally, 240 °C was maintained for 4 min, for a total program time of 30 min [20,21,22].

During the analysis, the GC–MS interface was kept at 200 °C, with the mass spectrometer in the electron ionization (EI) mode (70 eV) and related to instrument tuning, and the ion source was kept at 200 °C. Mass spectra were collected over 35 to 500 m/z in range in the total ion current (TIC) mode, with scan intervals at 0.3 s. VOC identification was carried out using the NIST11 and the FFNSC2 libraries of mass spectra.

Chromatogram peak integration was performed using the peak area as target parameter programming an automatic integration round using 70 as the minimum number of peak detection and 500 as the minimum area to detect. Other parameters used in the automatic peak integration were: slope 100/min, width 2 s, drift 0/min, doubling time (T.DBL) 1000 min, and no smoothing method was applied. The final round of peak integration was performed by manual peak integration for all the obtained chromatograms.

### 2.3. S3 Detection

**Figure 2:**
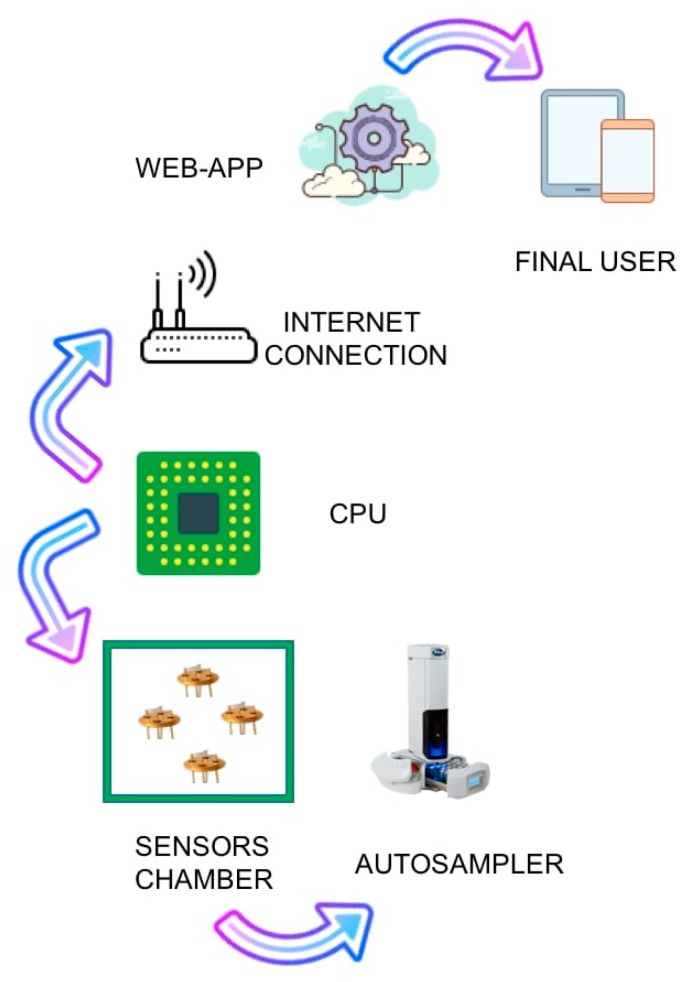
Diagram representing S3 device components.

S3 is an acronym for small sensor systems, which is a device developed by the collaboration of the groups involved in this study. S3 was previously used, with considerable success, in numerous studies applied to the field of food technology and quality control [23,24]. S3 consists of an electronic part to manage the signal, in order to send acquired data to the cloud, where it is possible to store and analyze them; an element that allows its connection to the internet; a pump to bring the volatile compounds to the heart of the instrument, as shown in Figure 2.

The volatile compounds collected by a pneumatic pump are conveyed inside an autosampler HT280T, supporting a 42-loading site carousel, (HTA s.r.l., Brescia, Italy), able to reduce the possible variables due to the preparation of the sample. The autosampler, used for the S3 device is the same model also used to prepare the samples seen in Section 2.2 associated with the GC–MS. S3 is equipped with an array of chemiresistor type sensors. The sensors grown and developed for this work were created with two techniques, Rheotaxial Growth Thermal Oxidation (RGTO) and nanowire technology. The RGTO technique involves two deposition steps: the first stage of a metallic thin film by DC magnetron sputtering from a metallic target on a substrate at higher temperatures than the melting point of the metal, then the thermal oxidation cycle in order to get a metal oxide layer with stable stoichiometry [25]. The surface of the thin film is rough, and this is an advantage since it provides a high surface-to-volume ratio and reactivity with gaseous species [26]. In addition, the existence of such very rough surface morphology, usually named ‘spongy agglomerates’, gives rise to a high specific area required for high-sensitivity gas sensors [27].

Nanowires exhibit exceptional crystalline quality and a very high length-to-width ratio, subsequent in enhanced sensitivity as well as long-term material stability for prolonged operation [28,29]. The experimental process consists of the evaporation of the powder (metal oxide) at high temperatures in a controlled atmosphere at pressures lower than hundreds of mbar (50–200 mbar) and the following mass transport of the vapor (50–100 sccm) towards the substrates kept at lower temperatures with respect to the source evaporation region. This growth technique is called the vapor–liquid–solid (VLS) mechanism. For SnO_2_ sensors, powders are positioned in the center of the furnace at 1370 °C, then an inert air flow at temperatures between 350 and 500 °C is used as carrier from furnace to substrates, where nanowires start growing.

Regarding CuO sensors, a thin layer of metallic copper was deposited on target substrates by RF magnetron sputtering, with a constant gas flow kept at 7 sccm. Copper is very reactive in environmental atmosphere, and the interaction with oxygen spontaneously produces a thin layer of copper oxide that must be removed to allow the growth of nanowires. After copper oxide layer removal, the sensors undergo a forced oxidation in a carbolite tubular furnace for 15 h [30].

The list of the 5 sensors produced at Sensor Lab and used for this study is presented in Table 2. The other 4 sensors were commercial sensors—MQ2, MQ3, TGS2611 and TGS2602.

In Table 3, a list of replicas analyzed for each sample is reported. Again, from the table, the number of total measures for each flavor is 45 for apricot, 34 for strawberry, and 37 for cherry.

### 2.4. Data Analysis Methods

From GC–MS analysis, a list of all the VOCs of the samples was found. The aim of this study was to find which compounds were in common for different recipes of the same flavor. Common VOCs were obtained comparing VOC lists obtained from GC–MS analysis, considering all the recipes for every single fruit. Only VOCs present in all the recipes were considered and shown in the following tables.

S3 analysis output consists of the resistance variation of the sensor due to VOC analysis. First, sensor responses in terms of resistance (Ω) were normalized when compared to the first value of the acquisition (R0). For all the sensors, the difference between the first value and the minimum value during the analysis time was calculated; hence, ΔR/R0 has been extracted as featured. For each taste, a specific matrix has been created: the rows contain all the measures for that taste considering all the recipes, the columns features are extracted from the sensors array.

Linear discriminant analysis (LDA) was applied to the three matrices built in this way. LDA is a well-known supervised technique for the dimensionality reduction of a dataset or classification purposes. Its aim is to minimize intra-class variability and to maximize separation between classes. Hence, LDA was used to determine whether the recipes could be distinguished by sensors. To calculate the accuracies of recipe recognition, venetian blinds was used as a cross validation method dividing each dataset in 10 folds.

## 3. Results and Discussion

In this section, the results obtained during this study will be presented. All the results obtained in the phase of the identification and analysis of volatile compounds (VCs) with GC–MS and SPME analysis will be used for the creation of the database and for training through artificial intelligence of the S3 device.

### 3.1. Determination of VOCs

The results obtained from the characterization of the jams of t three flavors and five analyzed recipes will be presented below. The results obtained are representative of three replicas for each of the recipes and flavors analyzed, for a total of:

3 flavor × 5 recipe × 3 replicas = 45 samples

All the compounds presented in the table were found in all three replicas of all the five recipes analyzed, belonging to the strawberry, apricot and cherry taste (Table 4, Table 5 and Table 6). The table below will highlight the individual contributions of the individual compounds with the characteristic smell of the analyzed jams. The abundance work shown in the table is obtained by averaging the three replicas of each sample.

The characteristic odor of fruits and of the production process is not influenced by the recipe and the raw materials used to obtain it. This also highlights the naturalness of the analyzed product, which does not compromise the real characteristics of fruits.

### 3.2. E-Nose Performance

**Figure 3:**
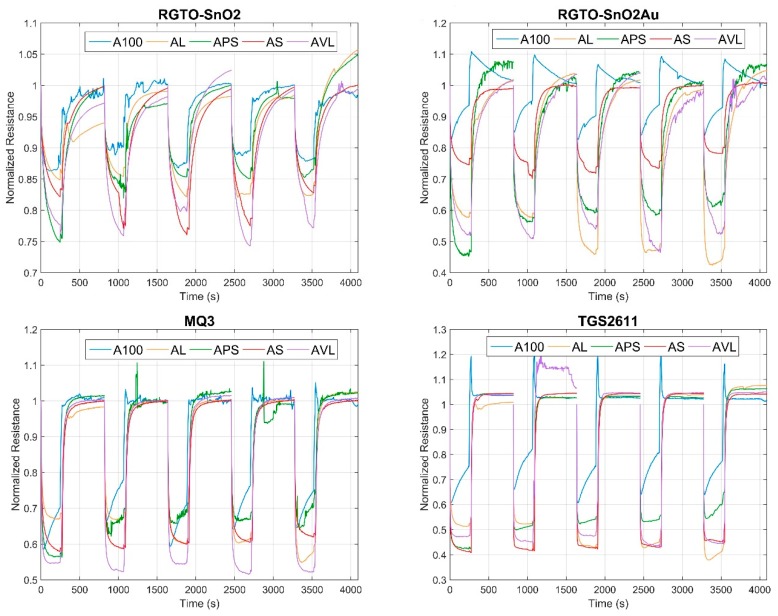
Signals obtained from four sensors: RGTO SnO_2_ (upper left side), RGTO SnO_2_Au (upper right side), MQ3 (low left side), and TGS2611 (low right side). Five measures are shown as an example of the typical sensor responses to apricot.

Chemical sensors used in this work have the peculiarity to change their resistance once they are exposed to pure gases or mixtures, such as VOCs. In Figure 3, the trends of four sensors from the array are shown for apricot measures and for all the recipes. In particular, all the graphs have time indication in seconds on the x-axis and on normalized resistance the y-axis; this is the reason why all signals start from a value equal to 1. Once sensing materials react with VOCs, resistance decreases. This reduction changes from sensor to sensor: indeed, each sensor reaches a different minimum resistance and the shape of the signals are diverse, too. Furthermore, focusing on a single sensor, it can be observed that sensors respond differently to the five recipes. Taking the RGTO-SnO_2_Au sensor as an example, the samples APS, AVL and AL are more similar in respect to A100 and AS. Otherwise, TGS2611 sensor signals show that A100 samples are more different from the other four types. This is the main reason why sensors arrays are used since metal oxide (MOX) sensors are non-specific sensors and respond to a broad range of molecules. In that way, it is possible to distinguish among the recipes taking into account information coming from different sources.

When VOC exposition ends, filtrated ambient air is fluxed to restore the sensor baseline. The baseline is recovered when resistance value returns close to the value 1, as shown in the normalized graphs of Figure 3. At the end of VOC analysis, the needle of the autosampler that aspires headspace sample comes out of the vial and this can produce an abrupt change of pressure in the flow that arrives in the sensor chamber. This can cause a sudden change in the response, as it can be observed for A100 measures in RGTO-SnO_2_Au and TGS2611 sensors. However, after the overshoot, signals tend to converge to the value of 1.

**Figure 4:**
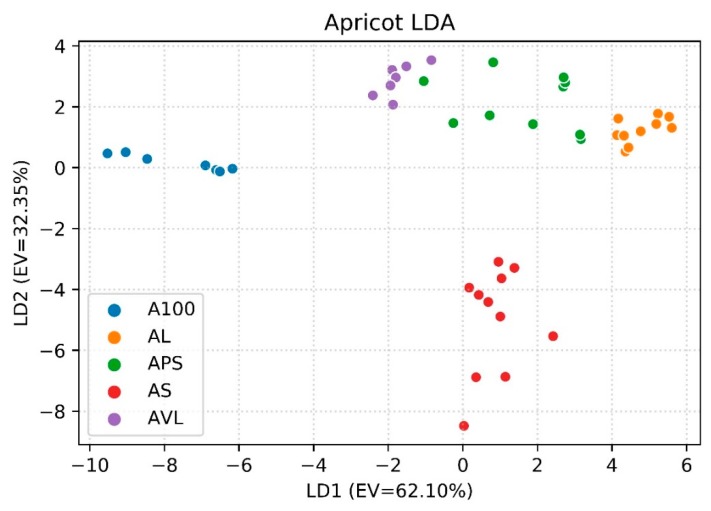
First two LD (linear discriminant) components for discrimination between the recipes of apricot jam (explained variance (EV) = 94.45%); LDA: linear discriminant analysis; A100, AL, APS, AS, AVL: sample code.

From signal observation, it has been decided that ΔR/R0 could be a feature suitable for our purposes. Three matrices have been built with these values—one for each fruit. LDA was applied to each dataset to determine how different classes (i.e., recipes) cluster. In Figure 4, the LDA graph with the first two linear discriminants (LDs) is shown, for a total explained variance (EV) equal to 94.45%. That means that almost all the information carried by features has been useful to discriminate between the classes. The graph shows that the A100 and AS samples are very different in respect to the others. On the contrary, the AL, APS and AVL clusters are closer but they do not overlap. This can be explained looking at the compounds obtained from the GC–MS analysis. Lists of all VOCs can be found in Appendix A (Table A1, Table A2, Table A3, Table A4, Table A5, Table A6, Table A7, Table A8, Table A9, Table A10, Table A11, Table A12, Table A13, Table A14 and Table A15). The quantities of common VOCs compared to the total of compounds considering each recipe varies from 64.65% to 76.33%. This difference does not explain the reason why the five groups cluster in the way shown in Figure 4. All recipes have furfural, α-terpineol, 1,6-octadien-3-ol, 3,7-dimethyl- and benzaldehyde among the first six most abundant compounds; these are also compounds that are present in all the recipes as shown in Table 4. The AS aromatic profile differs from the other because it has ammonium acetate (instead of acetic acid like the other recipes) and acetone among the compounds with the highest quantities, and a bigger area of the peak of benzoic acid (11.11% of relative abundance, while it is between 0.33% and 0.75% for the other four recipes). Regarding A100, it has 2H-pyran, 2-ethenyltetrahydro-2,6,6-trimethyl- (2.5%, in the other recipes is between 0.91% and 1.19%) naphthalene, and 1,2,3,4-tetrahydro-1,1,6-trimethyl- (2.2%, while it is between 0.37% and 0.48% for the others; it is absent in AL samples) within the top ten most present compounds. Calculation of LDA performances led to obtain an accuracy of 93.48%.

**Figure 5:**
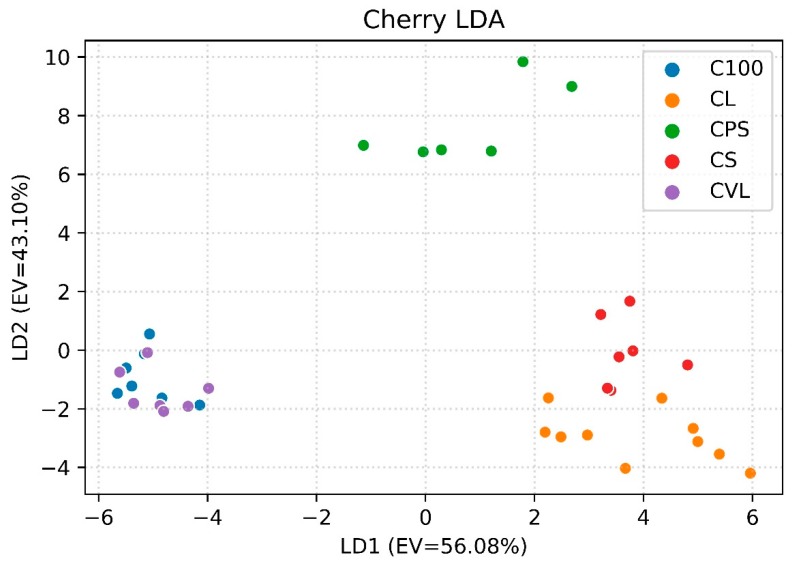
First two LD components for discrimination between the recipes of cherry jam (EV = 99.18%).

Figure 5 shows the same typology of graph for cherry samples (EV = 99.18%). Unlike what is shown in Figure 4, C100 and CVL clusters overlap; in this case, the array seems to not be able to recognize that those samples come from different recipes. The CL and CS groups are very close on the graph. For cherry samples, the relative abundance of common compounds is 4.12% for CPS, 43.79% and 53.32% for C100 and CVL respectively, and 80% and 85.28% for CL and CS, respectively. These values reflect how the different recipes cluster in the LDA graph, and the relative abundance of common compounds can explain this result in this case. Furthermore, CPS is the only recipe that contains D-limonene—the abundance of which is 43 times higher than the second most abundant compound of the recipe in terms of the area of the peak and has lower quantities of furfural (0.59%) and benzaldehyde (1.07%). Indeed, the C100 and CVL recipes have an amount of furfural equal to 16.2% and 28% and of benzaldehyde equal to 5.47% and 11.7% respectively. However, for the CL and CS samples, benzaldehyde (58.65% and 59.5%) is much more present than furfural (18.24% and 8.42%). In this case, LDA achieves a classification rate equal to 83.78%. This result is lower than the previous due to the overlap of C100 and CVL clusters and to the fact that CL and CS are not 100% linearly separable.

**Figure 6:**
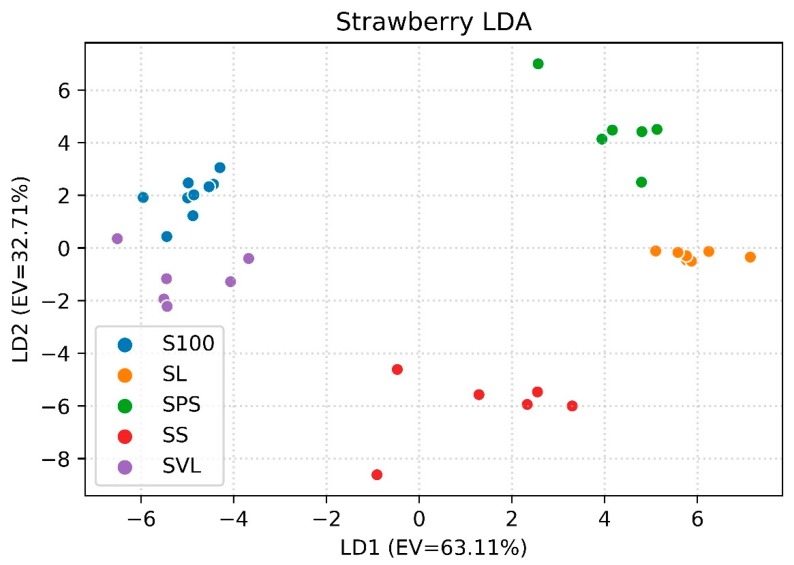
First two LD components for discrimination between the recipes of strawberry jam (EV = 95.82%).

Finally, in Figure 6, the LDAs for strawberry specimens are presented in the same way as for the previous fruits (EV = 95.82%). S100 and SVL are the closest groups—a result that can be compared to the one obtained with cherry. On the contrary, there is better distinction between SS and SL in respect to CS and CL. In addition to the apricot samples, strawberry specimens show similar values of the abundance of common VOCs; these percentages range from 54.12% to 75.01%. However, there are some differences if the most abundant compounds for each recipe are compared. The distance of the SS cluster from the others can be explained by the presence of acetone (relative abundance of 10.38%, which is absent in the remaining recipes), ethyl acetate (4.58%; it is present only in SL for a quantity equal to 2.48%) and benzoic acid (3.83%, in comparison to 0.35%–0.80% in the other samples) among the most abundant compounds. S100 and SVL have almost the same most abundant compounds (furfural, benzaldehyde, α-terpineol, 1,6-octadien-3-ol, 3,7-dimethyl-, ammonium acetate and pentanal), except for the presence of cyclobutane, 1,2-bis(1-methylethenyl)-, trans- (10.87% of relative abundance) and dimethyl sulfide (6,20%) in S100 and nerolidyl acetate (12.46%) in SVL. The same common VOCs can be found in SPS and SL (sample code); in this case, sensors recognition of the recipe could be addressed to 2-Heptenal, (Z)- (SPS: 3,6%; SL: not present), Acetophenone (SPS: 3,39%; SL: 1.49%), Butanoic acid, 2-methyl- (SPS: 1,52%; SL: 2.67%) and Ethanone, 1-(2-furanyl)- (SPS: 1.53%; SL: 2.48%). Nevertheless, LDA accuracy is higher among the three recipes and it is equal to 97.06%.

## 4. Conclusions

This study aimed to characterize the aromatic fingerprint of fruit jams, taking into account three flavors and five recipes for a total of 15 different samples. The sets of VOCs provided by the GC–MS analysis showed that each specimen has a unique aroma, although there are common compounds among the recipes for each fruit. As derived from literature, those VOCs come from the common base that is the fruit itself. Hence, it is possible to assert that the thermal processes necessary for jam production do not alter the content of the used fruit. It is interesting to notice that all the samples have a small set of VOCs that has always been detected in this study: furfural; benzaldehyde; 1,6-Octadien-3-ol, 3,7-dimethyl-; L-α-Terpineol; benzoic acid.

Furthermore, the innovative and real-time method developed for this study using the S3 device has excellent characteristics to be used in the production of this type of food product. In fact, once trained, S3 manages to highlight the real product differences and to provide a data item in a few minutes. Hence, the system provides an important information to promote and increase the guarantee of high standards of natural products. The impact of the work in this subject will be remarkable due to the lack of a reported bibliography. This work will pave the way to new studies that will deal with complex matrices as fruit jams and marmalades.

## Figures and Tables

**Table 1:** List of samples analyzed: 3 flavors and 5 recipes for each taste.

Taste	Sample Code	Ingredients
Apricot	APS	Apricots; grape sugar; gelling agent: pectin; acidity correctors: citric acid and calcium citrate. Fruit used 70 g per 100 g.
	AL	Apricots; apple sugar; gelling agent: pectin; acidity corrector: calcium citrate. Fruit used 50 g per 100 g.
	AS	Apricots; fructose; water; gelling agent: pectin; acidity corrector: citric acid; calcium citrate; sweetener: steviol glycosides. Fruit used: 50 g per 100 g.
	A100	Apricots; grape sugar; concentrated lemon juice; gelling agent: fruit pectin. Used apricots: 100g per 100 g of product.
	AVL	Apricots; sugar; gelling agent: pectin; acidity correctors: citric acid and calcium citrate; sweetener: steviol glycosides. Fruit used 70 g per 100 g.
Strawberry	SPS	Strawberry; grape sugar; gelling agent: pectin; acidity corrector: citric acid; concentrated elderberry juice. Fruit used 70 g per 100 g.
	SL	Strawberries; apple sugar; gelling agent: pectin; acidity corrector: citric acid and calcium citrate; red fruit juice; concentrated elderberry juice. Fruit used 50 g per 100 g.
	SS	Strawberries; fructose; water; gelling agent: pectin; acidity corrector: citric acid and calcium citrate; concentrated elderberry juice; sweetener: steviol glycosides. Fruit used: 50 g per 100 g.
	S100	Strawberries; grape sugar; gelling agent: pectin; concentrated lemon juice; concentrated elderberry juice. Used strawberries: 100 g per 100 g of product.
	SVL	Strawberries; sugar; gelling agent: pectin; acidity correctors: citric acid and calcium citrate; concentrated elderberry juice; sweetener: steviol glycosides. Fruit used 70 g per 100 g.
Cherry	CPS	Cherries; sugar; concentrated lemon juice; gelling agent: pectin; acidity corrector: calcium citrate. Fruit used 50 g per 100 g of product.
	CL	Cherries; apple sugar; gelling agent: pectin; acidity correctors: citric acid and calcium citrate. Fruit used 50 g per 100 g.
	CS	Cherries; fructose; water; gelling agent: pectin; acidity corrector: citric acid and calcium citrate; sweetener: steviol glycosides. Fruit used: 50 g per 100 g.
	C100	Cherries; grape sugar; concentrated lemon juice; gelling agent: fruit pectin. Used cherries: 100 g per 100 g of product.
	CVL	Cherries; sugar; gelling agent: pectin; acidity correctors: citric acid and calcium citrate; sweetener: steviol glycosides. Fruit used 70 g per 100 g.

**Table 2:** List of sensors produced at Sensors Lab, University of Brescia.

Materials	Morphology	Working Temperature (°C)
SnO_2_ + Au	RGTO	400
SnO_2_	RGTO	400
CuO	Nanowire	350
SnO_2_ + Au	Nanowire	350
SnO_2_	Nanowire	350

RGTO, Rheotaxial Growth Thermal Oxidation.

**Table 3:** List of samples analyzed with an S3 device, divided for the different recipes.

Taste	Sample Code	Number of Replicas for S3
Apricot	APS	10
	AL	10
	AS	11
	A100	7
	AVL	7
Strawberry	SPS	6
	SL	7
	SS	6
	S100	9
	SVL	6
Cherry	CPS	6
	CL	10
	CS	7
	C100	7
	CVL	7

**Table 4:** List of the compounds identified in all the strawberry recipes.

Retention Time	Compound	SPS	SVL	S100	SL	SS	Description
11.343	Acetoin	5.90 × 10^5^	9.62 × 10^5^	6.90 × 10^5^	7.36 × 10^5^	1.40 × 10^6^	Pleasant, buttery odor. Acetoin is used as a food flavoring (in baked goods) and as a fragrance. It can be found in apples, butter, yogurt, asparagus, blackcurrants, blackberries, wheat, broccoli, brussels sprouts, cantaloupes and maple [31].
14.527	Linalool oxide	6.66 × 10^5^	8.44 × 10^5^	1.72 × 10^6^	7.97 × 10^5^	1.23 × 10^6^	Furanoid with floral odor type, and musty, camphoreous, herbal, balsamic, and pine notes. Other name: 2-(5-ethenyl-5-methyloxolan-2-yl) propan-2-yl ethyl carbonate (IUPAC) [32].
14.689	Ammonium acetate	6.16 × 10^6^	6.77 × 10^6^	7.92 × 10^6^	4.18 × 10^6^	6.22 × 10^6^	Slight odor of acetic acid. Its use as a food additive regulator of acidity is authorized in Australia and New Zealand, where it is identified by the number INS 264 and in the European Union where it is identified by the code E264 [33].
14.931	Furfural	2.27 × 10^7^	1.07 × 10^7^	1.88 × 10^7^	1.23 × 10^7^	1.35 × 10^7^	The compound is an aldehyde group attached to the 2-position of furan. It is a product of the acid catalyzed dehydration of five-carbon sugars (pentoses), particularly xylose. These sugars may be obtained from hemicellulose present in lignocellulosic biomass, which can be extracted from most terrestrial plants. It is found in many foods: coffee (55–255 mg/kg) and whole-grain bread (26 mg/kg). When heated in the presence of acids, furfural irreversibly polymerizes, acting as a thermosetting polymer [34].
15.585	Ethanone and 1-(2-furanyl)-	1.94 × 10^6^	1.08 × 10^6^	1.67 × 10^6^	1.48 × 10^6^	9.83 × 10^5^	It is found in alcoholic beverages. It is present in cooked apples, marasca cherry, wine grapes, peach, strawberry, plum, blueberry, asparagus, kohlrabi, baked potatoes, pineapple, bread products, rice, yogurt, wines, soy, and black tea. It contributes to the aroma of many foods and beverages. It is used in aromatic compositions. Another name: 2-acetylfuran [35].
15.893	Benzaldehyde	4.65 × 10^6^	3.64 × 10^6^	1.55 × 10^7^	1.24 × 10^7^	2.34 × 10^7^	Benzaldehyde has a role as a flavoring agent, fragrance and a plant metabolite. Benzaldehyde can be derived from natural sources and is colorless liquid that turns to brown on exposure to air. It is an aromatic aldehyde that carries a single formyl group with an almond smell. Benzaldehyde was first extracted from bitter almonds [36].
16.159	1,6-octadien-3-ol and 3,7-dimethyl-	1.50 × 10^7^	9.20 × 10^6^	9.33 × 10^6^	2.35 × 10^6^	3.87 × 10^6^	Known as linalool, it is a monoterpene abundantly present in the essence of rosewood. It is also found free or combined in the natural essential oils of coriander, basil, lavender or bergamot. It has an antimicrobial effect and it is present in the total pull of volatile organic compounds (VOCs) of many fruits and flowers. It is confirmed that linalool is the most important compound for strawberry flavor [37,38].
16.921	3(2H)-furanone and 4-methoxy-2,5-dimethyl-	2.56 × 10^6^	2.39 × 10^6^	2.25 × 10^6^	9.20 × 10^5^	6.99 × 10^5^	Furanone methyl ether typical of strawberries. Nice, caramelic, sweet moldy mushroom, vegetable potato, burnt sugar, nut skin, wasabi, fruity, brandy with cocoa and coffee notes [39].
17.769	Acetophenone	4.31 × 10^6^	6.67 × 10^5^	1.52 × 10^6^	8.89 × 10^5^	1.21 × 10^6^	It is the simplest aromatic ketone and an ingredient in fragrances that resemble almond, cherry, honeysuckle, jasmine, and strawberry. It is used in chewing gum. It is also listed as an approved excipient by the U.S. Food and Drug Administration (FDA) [40].
17.944	Butanoic acid and 2-methyl-	1.94 × 10^6^	2.87 × 10^6^	3.27 × 10^6^	1.59 × 10^6^	1.78 × 10^6^	(S)-2-Methylbutyric acid has a pleasantly sweet, fruity odor. (R)-2-methylbutanoic acid has a pervasive, cheesy, and sweaty odor [41].
18.305	L-α-terpineol	4.53 × 10^6^	4.02 × 10^6^	1.15 × 10^7^	1.47 × 10^6^	2.98 × 10^6^	α-Terpineol is a monoterpene alcohol that has been isolated from a variety of sources such as cajuput oil, pine oil, and petitgrain oil. It has a characteristic lilac odor, with a sweet taste reminiscent of peach on dilution. It is found in the composition of various essential oils [39,42].
20.141	Heptanoic acid	2.58 × 10^6^	2.85 × 10^6^	3.32 × 10^6^	1.24 × 10^6^	2.65 × 10^5^	It makes part of flavoring agents and related substances. Cheese and other dairy-type flavors, and ripe fruit especially apple and strawberry [43].
20.573	Benzyl alcohol	3.59 × 10^5^	6.02 × 10^5^	2.44 × 10^6^	2.85 × 10^5^	5.73 × 10^5^	Benzyl alcohol is an aromatic alcohol and a colorless liquid with a mild pleasant aromatic odor. It is a component of some essential oils such as jasmine, neroli, violet and ylang-ylang. It can be used as a preservative [44].
20.975	Phenylethyl alcohol	5.90 × 10^5^	2.00 × 10^5^	4.36 × 10^5^	2.22 × 10^5^	4.96 × 10^5^	Also known as 2-phenylethanol, it is an organic compound that consists of a phenethyl group attached to an OH group. It is a colorless liquid that is widely available in nature, found in a variety of essential oils. It has a pleasant floral odor [45].
22.531	Octanoic acid	3.00 × 10^5^	2.40 × 10^5^	2.87 × 10^5^	4.04 × 10^5^	3.98 × 10^5^	Octanoic acid is a volatile organic acid detected in strawberry jam at a conc of 2.9 mg/kg. [44,46].
23.573	2(3H)-furanone and 5-hexyldihydro-	2.78 × 10^6^	3.14 × 10^6^	3.30 × 10^6^	7.91 × 10^5^	1.06 × 10^6^	A furan compound linked to a ketone group with a fruity peach flavor [40].
26.429	Benzoic acid	4.52 × 10^5^	7.12 × 10^5^	7.84 × 10^5^	3.39 × 10^5^	4.74 × 10^6^	A simple aromatic carboxylic acid that occurs naturally in many plants and serves as an intermediate in the biosynthesis of many secondary metabolites, and it is found in post-harvested strawberries up to 29 mg/kg [47].

SPS, SVL, S100, SL, SS: sample code.

**Table 5:** List of the compound identified in all the apricot recipes.

Retention Time	Compound	APS	AVL	A100	AL	AS	Description
6.130	Hexanal	2.53 × 10^5^	1.99 × 10^6^	8.99 × 10^5^	8.03 × 10^5^	2.12 × 10^6^	Also called hexanaldehyde or caproaldehyde, it is an alkyl aldehyde. Its scent resembles freshly cut grass, with a powerful, penetrating characteristic fruity odor and taste. It occurs naturally and contributes a hay-like “off-note” flavor in green peas [21,39,48].
6.707	Linalool oxide	1.21 × 10^6^	9.72 × 10^5^	5.27 × 10^6^	7.54 × 10^5^	1.56 × 10^6^	Furanoid with floral odor type, with musty, camphoreous, herbal, balsamic, and pine notes. Linaloil oxide is found in alcoholic beverages. It is an aromatic and fragrant ingredient. Linaloile oxide is present in roselle tea, muscat grapes, lime oil, alfalfa, Riesling wine, grapefruit, yellow passion fruit, apricot, blackberry, blueberry, and nectarine. Other name: 2-(5-ethenyl-5-methyloxolan-2-yl) propan-2-yl ethyl carbonate (IUPAC) [32].
11.964	Cyclohexanone, 2,2,6-trimethyl-	5.02 × 10^5^	4.54 × 10^5^	8.28 × 10^5^	4.63 × 10^5^	9.01 × 10^5^	Found in bilberries, passion fruit and tea. Also found in apricot, bilberry, white wine, black tea, green tea, microbial fermented tea, brewed tea, yellow passion fruit juice and dill herb [40].
12.523	5-Hepten-2-one, 6-methyl-	1.37 × 10^6^	1.69 × 10^6^	2.13 × 10^6^	1.25 × 10^6^	1.84 × 10^6^	Also known as sulcatone, it is an unsaturated methylated ketone with a citrus-like, fruity odor. Sulcatone is one of a number of mosquito attractants and has been also found in apricot scent [39,49].
14.931	Furfural	3.52 × 10^7^	1.46 × 10^7^	5.87 × 10^7^	2.40 × 10^7^	4.37 × 10^7^	The compound is an aldehyde group attached to the 2-position of furan. It is a product of the acid catalyzed dehydration of five-carbon sugars (pentoses), particularly xylose. These sugars may be obtained from hemicellulose present in lignocellulosic biomass, which can be extracted from most terrestrial plants. It is found in many foods: coffee (55–255 mg/kg) and whole-grain bread (26 mg/kg). When heated in the presence of acids, furfural irreversibly polymerizes, acting as a thermosetting polymer [34,39].
15.881	Benzaldehyde	3.08 × 10^6^	2.64 × 10^6^	5.58 × 10^6^	3.03 × 10^6^	7.65 × 10^6^	A colorless liquid that turns to brown on exposure to air. Benzaldehyde has a characteristic odor and aromatic taste, similar to bitter almond, and is also produced during the ripening of apricots [39,50].
16.143	1,6-Octadien-3-ol, 3,7-dimethyl-	3.03 × 10^7^	2.66 × 10^7^	3.27 × 10^7^	3.80 × 10^6^	4.83 × 10^6^	Known as linalool, it is a monoterpene abundantly present in the essence of rosewood. It is also found free or combined in the natural essential oils of coriander, basil, lavender or bergamot. It has an antimicrobial effect and it is present in the total pull of volatile organic compounds (VOCs) of many flowers and fruits such as apricots [39,48].
16.605	2-Furancarboxaldehyde, 5-methyl-	8.24 × 10^5^	2.51 × 10^5^	1.56 × 10^6^	5.97 × 10^5^	1.42 × 10^6^	Organic compounds known as aryl-aldehydes; it contains an aldehyde group directly attached to an aromatic ring. It is found in pepper (c. annuum). The 5-methyl-2-furancarboxaldehyde is isolated from brown algae and other plant sources, doubtless as a secondary produced from saccharides [39,48,50].
16.974	3-Cyclohexen-1-ol, 4-methyl-1-(1-methylethyl)-, (R)-	6.27 × 10^5^	1.13 × 10^6^	1.35 × 10^6^	6.28 × 10^5^	8.61 × 10^5^	It is also known as 4-terpineol and is a flavoring ingredient found in the aroma of apricots. It has a role as a plant metabolite, antibacterial agent, antioxidant, anti-inflammatory agent, and antiparasitic agent [51].
18.247	L-α-Terpineol	1.76 × 10^7^	1.97 × 10^7^	3.94 × 10^7^	4.16 × 10^6^	1.08 × 10^7^	α-Terpineol is a monoterpene alcohol that has been isolated from a variety of sources such as cajuput oil, pine oil, and petitgrain oil. It has a characteristic lilac odor with a sweet taste reminiscent of peach on dilution. It is found in the composition of various essential oils [39,42,51].
20.703	4-Acetyl-1-methylcyclohexene	2.59 × 10^5^	3.23 × 10^5^	7.88 × 10^5^	1.18 × 10^5^	2.21 × 10^5^	It is found in cereals and cereal products. The 4-Acetyl-1-methylcyclohexene is a flavoring ingredient. It is isolated from the famine food *Santalum album* (sandalwood) [48].
21.252	trans-.beta.-Ionone	9.88 × 10^5^	1.26 × 10^6^	1.44 × 10^6^	8.17 × 10^5^	1.12 × 10^6^	β-Ionone has a characteristic violet-like odor, and is fruitier and woodier than α-ionone.
26.326	Benzoic acid	4.44 × 10^5^	7.11 × 10^5^	9.42 × 10^5^	2.70 × 10^5^	1.53 × 10^7^	It is a simple aromatic carboxylic acid that occurs naturally in many plants and serves as an intermediate in the biosynthesis of many secondary metabolites. It is found during the ripening of apricots [50] and in post-harvest strawberries up to 29 mg/kg [47].

APS, AVL, A100, AL, AS: sample code.

**Table 6:** List of the compound identified in all the cherry recipes.

Retention Time	Compound	CPS	CVL	C100	CL	CS	Description
13.630	Nonanal	1.18 × 10^6^	3.49 × 10^5^	2.11 × 10^5^	1.08 × 10^5^	1.90 × 10^5^	Nonanal has a strong, fatty odor, developing an orange and rose note on dilution. It has a fatty, citrus-like flavor [39,40,52].
14.943	Furfural	5.77 × 10^6^	9.86 × 10^6^	7.46 × 10^6^	1.51 × 10^7^	1.26 × 10^7^	The compound is an aldehyde group attached to the 2-position of furan. It is a product of the acid catalyzed dehydration of five-carbon sugars (pentoses), particularly xylose. These sugars may be obtained from hemicellulose present in lignocellulosic biomass, which can be extracted from most terrestrial plants. It is found in many foods: coffee (55–255 mg/kg) and whole-grain bread (26 mg/kg). When heated in the presence of acids, furfural irreversibly polymerizes, acting as a thermosetting polymer [34,39,40].
15.881	Benzaldehyde	1.04 × 10^7^	4.14 × 10^6^	2.52 × 10^6^	4.84 × 10^7^	8.93 × 10^7^	Benzaldehyde has a role as a flavoring agent, fragrance and a plant metabolite. It is an aromatic aldehyde that carries a single formyl group. Benzaldehyde can be derived from natural source and is colorless liquid that turns to brown on exposure to air. Benzaldehyde has a characteristic odor and aromatic taste, similar to bitter almond, and is also produced during the ripening of apricots and cherries [36,39,50,52].
16.155	1,6-Octadien-3-ol, 3,7-dimethyl-	1.87 × 10^6^	2.42 × 10^6^	1.83 × 10^6^	1.05 × 10^6^	1.18 × 10^6^	Known as linalool, it is a monoterpene abundantly present in the essence of rosewood. It is also found free or combined in the natural essential oils of coriander, basil, lavender or bergamot. It has an antimicrobial effect and is present in the total pull of VOCs of many flowers and fruits as apricots and cherries [39,48,52].
18.264	L-α-Terpineol	2.01 × 10^7^	1.07 × 10^6^	7.82 × 10^6^	1.20 × 10^6^	1.63 × 10^6^	α-Terpineol is a monoterpene alcohol that has been isolated from a variety of sources such as cajuput oil, pine oil, and petitgrain oil. It has a characteristic lilac odor, with a sweet taste reminiscent of peach on dilution. It is found in the composition of various essential oils [39,42,51,52].
26.307	Benzoic acid	5.70 × 10^5^	5.87 × 10^5^	3.24 × 10^5^	2.09 × 10^5^	2.30 × 10^6^	It is a simple aromatic carboxylic acid that occurs naturally in many plants and serves as an intermediate in the biosynthesis of many secondary metabolites. It is found during the ripening of apricots [50] and in post-harvest strawberries up to 29 mg/kg [47].

CPS, CVL, C100, CL, CS: sample code.

## Appendix A

**Table A1:** APS sample VOCs.

Retention Time	Name	Mean
1.655	l-Alanine ethylamide, (S)-	1.72 × 10^5^
2.363	Acetone	8.67 × 10^4^
2.980	1,3-Propanediol, diacetate	1.25 × 10^6^
4.136	Pentanal	4.27 × 10^6^
6.103	Hexanal	2.53 × 10^5^
6.707	2H-Pyran, 2-ethenyltetrahydro-2,6,6-trimethyl-	1.21 × 10^6^
9.000	Cyclohexene, 1-methyl-5-(1-methylethenyl)-, (R)-	2.22 × 10^5^
9.150	Eucalyptol	1.76 × 10^5^
9.455	4-Ethylbenzoic acid, 1-(cyclopentyl)ethyl ester	1.68 × 10^5^
10.310	5-Decen-1-ol, (E)-	4.43 × 10^5^
10.502	1-Pentanol	4.96 × 10^5^
11.248	(+)-4-Carene	2.92 × 10^5^
11.319	Acetoin	1.97 × 10^5^
11.717	1-Butanol, 2-methyl-, acetate	3.27 × 10^5^
11.786	Hexane, 3-ethyl-	6.91 × 10^4^
11.964	Cyclohexanone, 2,2,6-trimethyl-	5.02 × 10^5^
12.200	2-Buten-1-ol, 3-methyl-	4.76 × 10^5^
12.305	2,5-Octanedione	3.58 × 10^5^
12.523	5-Hepten-2-one, 6-methyl-	1.37 × 10^6^
13.261	Cyclopropane, trimethyl(2-methyl-1-propenylidene)-	5.67 × 10^4^
13.682	Nonanal	1.62 × 10^5^
13.748	1H-Pyrazole, 4,5-dihydro-5,5-dimethyl-4-isopropylidene-	4.91 × 10^5^
13.885	Propanenitrile, 3-(1-methylethoxy)-	1.02 × 10^5^
14.000	Ethane, 1,2-bis[(4-amino-3-furazanyl)oxy]-	1.34 × 10^5^
14.148	Benzene, 1-cyclopropylmethyl-4-(1-methylethyl)-	1.08 × 10^5^
14.194	Benzene, 1-cyclohexyl-3-methyl-	4.04 × 10^4^
14.255	2-Octenal, (E)-	1.75 × 10^5^
14.465	α-Methyl-α-[4-methyl-3-pentenyl]oxiranemethanol	1.55 × 10^6^
14.527	Ethyl 2-(5-methyl-5-vinyltetrahydrofuran-2-yl)propan-2-yl carbonate	5.00 × 10^5^
14.689	Acetic acid	6.19 × 10^6^
14.931	Furfural	3.52 × 10^7^
15.244	1-Hexanol, 2-ethyl-	2.50 × 10^5^
15.573	Ethanone, 1-(2-furanyl)-	1.72 × 10^6^
15.881	Benzaldehyde	3.08 × 10^6^
16.143	1,6-Octadien-3-ol, 3,7-dimethyl-	3.03 × 10^7^
16.286	Naphthalene, 1,2,3,4-tetrahydro-1,1,6-trimethyl-	6.42 × 10^5^
16.605	2-Furancarboxaldehyde, 5-methyl-	8.24 × 10^5^
16.838	Bicyclo[6.1.0]nonane, 9,9-dichloro-	1.24 × 10^5^
16.974	3-Cyclohexen-1-ol, 4-methyl-1-(1-methylethyl)-, (R)-	6.27 × 10^5^
17.049	5,7-Octadien-2-ol, 2,6-dimethyl-	5.47 × 10^5^
17.272	1-Cyclohexene-1-carboxaldehyde, 2,6,6-trimethyl-	1.44 × 10^6^
17.441	2(5H)-Furanone, 4,5,5-trimethyl-3-(3-methyl-2-methylenebutyl)-	2.03 × 10^6^
17.502	1-methyl-4-(prop-1-en-2-yl)-7-oxabicyclo[4.1.0]heptan-2one	4.42 × 10^5^
17.770	2-Furanmethanol	5.34 × 10^5^
17.896	Butanoic acid, 2-methyl-	1.80 × 10^6^
18.247	L-α-Terpineol	1.76 × 10^7^
18.383	Hexanoic acid, anhydride	8.31 × 10^5^
18.789	2,6-Octadiene, 1-(1-ethoxyethoxy)-3,7-dimethyl-	2.59 × 10^5^
18.968	1, 1, 5-Trimethyl-1, 2-dihydronaphthalene	1.14 × 10^6^
19.276	Dodecanedioic acid, bis(tert-butyldimethylsilyl) ester	5.03 × 10^4^
20.067	Cyclopentaneundecanoic acid	2.80 × 10^6^
20.226	5,9-Undecadien-2-one, 6,10-dimethyl-, (Z)-	8.05 × 10^5^
20.366	7-Octen-3-ol, 2,3,6-trimethyl-	5.37 × 10^4^
20.504	Benzyl alcohol	3.60 × 10^5^
20.703	4-Acetyl-1-methylcyclohexene	2.59 × 10^5^
20.911	Phenylethyl Alcohol	3.78 × 10^5^
21.252	trans-β-Ionone	9.88 × 10^5^
21.433	Bromoacetic acid, dodecyl ester	3.20 × 10^5^
21.790	4-Hexen-1-ol, 6-(2,6,6-trimethyl-1-cyclohexenyl)-4-methyl-, (E)-	1.93 × 10^5^
21.956	Carbamic acid, phenyl ester	1.90 × 10^5^
22.244	Nerolidyl acetate	1.32 × 10^6^
22.300	1,6,10-Dodecatrien-3-ol, 3,7,11-trimethyl-, [S-(Z)]-	1.34 × 10^5^
22.461	Octanoic acid	2.22 × 10^5^
23.517	2(3H)-Furanone, 5-hexyldihydro-	7.04 × 10^5^
25.625	2(4H)-Benzofuranone, 5,6,7,7a-tetrahydro-4,4,7a-trimethyl-	4.59 × 10^5^
25.780	gamma.-Dodecalactone	5.03 × 10^4^
26.326	Benzoic acid	4.44 × 10^5^
26.615	Tridecanoic acid	6.65 × 10^4^
26.890	5-Hydroxymethylfurfural	1.52 × 10^5^
26.973	2-tert-Butyl-4-hexylphenol	4.69 × 10^4^
27.331	1,2-Benzenedicarboxylic acid, bis(2-methylpropyl) ester	2.48 × 10^5^
27.729	di(Butoxyethyl)adipate	2.41 × 10^5^
28.857	Phosphine, 1,3-propanediylbis[bis(1-methylethyl)-	6.88 × 10^4^

**Table A2:** AVL sample VOCs.

Retention Time	Name	Mean
2.390	2-Hexanone, 4-hydroxy-5-methyl-3-propyl-	2.07 × 10^5^
6.146	Hexanal	1.99 × 10^6^
6.703	2H-Pyran, 2-ethenyltetrahydro-2,6,6-trimethyl-	9.72 × 10^5^
10.333	9-Dodecen-1-ol, acetate, (Z)-	3.72 × 10^5^
11.344	Acetoin	3.12 × 10^5^
11.990	Cyclohexanone, 2,2,6-trimethyl-	4.54 × 10^5^
12.214	2-Heptenal, (Z)-	4.12 × 10^5^
12.544	5-Hepten-2-one, 6-methyl-	1.69 × 10^6^
13.644	Nonanal	3.00 × 10^5^
13.769	1H-Pyrazole, 4,5-dihydro-5,5-dimethyl-4-isopropylidene-	9.69 × 10^5^
14.037	1,3-Hexadiene, 3-ethyl-2-methyl-	3.06 × 10^5^
14.279	2-Octenal, (E)-	3.51 × 10^5^
14.485	α-Methyl-α-[4-methyl-3-pentenyl]oxiranemethanol	2.01 × 10^6^
14.711	Acetic acid	5.22 × 10^6^
14.951	Furfural	1.46 × 10^7^
15.264	1-Hexanol, 2-ethyl-	2.11 × 10^5^
15.378	2,4-Heptadienal, (E,E)-	1.14 × 10^5^
15.903	Benzaldehyde	2.64 × 10^6^
16.160	1,6-Octadien-3-ol, 3,7-dimethyl-	2.66 × 10^7^
16.365	Naphthalene, 1,2,3,4-tetrahydro-1,1,6-trimethyl-	3.91 × 10^5^
16.630	2-Furancarboxaldehyde, 5-methyl-	2.51 × 10^5^
16.766	3-Buten-2-ol, 4-(2,6,6-trimethyl-1-cyclohexen-1-yl)-	1.80 × 10^5^
16.994	3-Cyclohexen-1-ol, 4-methyl-1-(1-methylethyl)-, (R)-	1.13 × 10^6^
17.292	1-Cyclohexene-1-carboxaldehyde, 2,6,6-trimethyl-	1.82 × 10^6^
17.461	1-methyl-4-(prop-1-en-2-yl)-7-oxabicyclo[4.1.0]heptan-2one	1.11 × 10^6^
17.913	Butanoic acid, 2-methyl-	9.28 × 10^5^
18.266	L-α-Terpineol	1.97 × 10^7^
19.006	1, 1, 5-Trimethyl-1, 2-dihydronaphthalene	7.98 × 10^5^
19.754	Bicyclo[3.1.0]hexane-6-methanol, 2-hydroxy-1,4,4-trimethyl-	1.25 × 10^5^
20.088	Cyclopentaneundecanoic acid	2.59 × 10^6^
20.245	5,9-Undecadien-2-one, 6,10-dimethyl-, (Z)-	1.11 × 10^6^
20.523	Benzyl alcohol	4.97 × 10^5^
20.719	4-Acetyl-1-methylcyclohexene	3.23 × 10^5^
20.933	Phenylethyl Alcohol	2.65 × 10^5^
21.276	trans-β-Ionone	1.26 × 10^6^
21.990	4-(2,4,4-Trimethyl-cyclohexa-1,5-dienyl)-but-3-en-2-one	1.69 × 10^5^
22.262	1,6,10-Dodecatrien-3-ol, 3,7,11-trimethyl-, [S-(Z)]-	1.92 × 10^5^
22.482	Octanoic acid	1.83 × 10^5^
23.537	2(3H)-Furanone, 5-hexyldihydro-	4.82 × 10^5^
25.648	2(4H)-Benzofuranone, 5,6,7,7a-tetrahydro-4,4,7a-trimethyl-	6.93 × 10^5^
26.342	Benzoic acid	7.11 × 10^5^
26.630	Tridecanoic acid	1.04 × 10^5^

**Table A3:** A100 sample VOCs.

Retention Time	Name	Mean
4.129	Pentanal	4.47 × 10^6^
6.164	Hexanal	8.99 × 10^5^
6.723	2H-Pyran, 2-ethenyltetrahydro-2,6,6-trimethyl-	5.27 × 10^6^
9.027	Cyclobutane, 1,2-bis(1-methylethenyl)-, trans-	3.34 × 10^6^
9.452	Z-7-Decen-1-yl acetate	1.68 × 10^6^
10.333	Bicyclo[3.1.0]hexane-6-methanol, 2-hydroxy-1,4,4-trimethyl-	2.46 × 10^6^
11.274	(+)-4-Carene	1.05 × 10^6^
11.729	2-Propanone, 1-hydroxy-	5.09 × 10^5^
11.995	Cyclohexanone, 2,2,6-trimethyl-	8.28 × 10^5^
12.325	2,5-Octanedione	5.20 × 10^5^
12.554	5-Hepten-2-one, 6-methyl-	2.13 × 10^6^
13.284	1,5,5-Trimethyl-6-methylene-cyclohexene	3.09 × 10^5^
13.654	Nonanal	5.36 × 10^5^
13.775	Isophorone	7.89 × 10^5^
14.169	(1,4-Dimethylpent-2-enyl)benzene	1.53 × 10^6^
14.494	Ethyl 2-(5-methyl-5-vinyltetrahydrofuran-2-yl)propan-2-yl carbonate	5.08 × 10^6^
14.712	Acetic acid	4.14 × 10^6^
14.958	Furfural	5.87 × 10^7^
15.273	1-Hexanol, 2-ethyl-	1.43 × 10^6^
15.602	Ethanone, 1-(2-furanyl)-	2.85 × 10^6^
15.853	Benzaldehyde	5.58 × 10^6^
16.169	1,6-Octadien-3-ol, 3,7-dimethyl-	3.27 × 10^7^
16.326	Naphthalene, 1,2,3,4-tetrahydro-1,1,6-trimethyl-	4.60 × 10^6^
16.644	2-Furancarboxaldehyde, 5-methyl-	1.56 × 10^6^
16.732	Megastigma-4,6(E),8(Z)-triene	5.55 × 10^5^
16.999	3-Cyclohexen-1-ol, 4-methyl-1-(1-methylethyl)-, (R)-	1.35 × 10^6^
17.075	7-Octen-2-ol, 2-methyl-6-methylene-	1.31 × 10^6^
17.303	1-Cyclohexene-1-carboxaldehyde, 2,6,6-trimethyl-	5.52 × 10^6^
17.474	3-Buten-2-one, 3-methyl-4-(1,3,3-trimethyl-7-oxabicyclo[4.1.0]heptan-1-yl)-	2.22 × 10^6^
17.791	2-Furanmethanol	6.00 × 10^5^
17.968	Benzene, 1,2,3,4-tetramethyl-4-(1-methylethenyl)-	1.47 × 10^6^
18.014	L-α-Terpineol	3.94 × 10^7^
18.791	Dodecanedioic acid, bis(tert-butyldimethylsilyl) ester	2.13 × 10^5^
18.999	1, 1, 5-Trimethyl-1, 2-dihydronaphthalene	3.89 × 10^6^
20.095	Geraniol	2.88 × 10^6^
20.257	5,9-Undecadien-2-one, 6,10-dimethyl-, (Z)-	7.75 × 10^5^
20.526	2-Caren-10-al	7.88 × 10^5^
20.731	4-Acetyl-1-methylcyclohexene	7.88 × 10^5^
20.942	Phenylethyl Alcohol	5.97 × 10^5^
21.285	trans-β-Ionone	1.44 × 10^6^
21.808	Calarene epoxide	6.02 × 10^5^
23.545	2(3H)-Furanone, 5-hexyldihydro-	7.06 × 10^5^
26.346	Benzoic acid	9.42 × 10^5^

**Table A4:** AL sample VOCs.

Retention Time	Name	Mean
2.389	Acetone	5.95 × 10^5^
2.978	Ethyl Acetate	2.50 × 10^6^
4.160	Pentanal	8.99 × 10^5^
6.176	Hexanal	8.03 × 10^5^
6.775	2H-Pyran, 2-ethenyltetrahydro-2,6,6-trimethyl-	7.54 × 10^5^
9.439	1-Butanol, 3-methyl-	4.50 × 10^5^
10.384	3-Tridecene	3.94 × 10^5^
11.780	5,9-Dodecadien-2-one, 6,10-dimethyl-, (E,E))-	2.58 × 10^5^
12.052	Cyclohexanone, 2,2,6-trimethyl-	4.63 × 10^5^
12.267	2-Heptenal, (Z)-	3.15 × 10^5^
12.377	2,5-Hexanedione	2.04 × 10^5^
12.599	5-Hepten-2-one, 6-methyl-	1.25 × 10^6^
13.816	Isophorone	6.51 × 10^5^
14.092	1,3-Hexadiene, 3-ethyl-2-methyl-	1.77 × 10^5^
14.324	2-Octenal, (E)-	9.62 × 10^4^
14.529	Ethyl 2-(5-methyl-5-vinyltetrahydrofuran-2-yl)propan-2-yl carbonate	1.35 × 10^6^
14.773	Acetic acid	3.04 × 10^6^
14.999	Furfural	2.40 × 10^7^
15.644	Ethanone, 1-(2-furanyl)-	1.31 × 10^6^
15.947	Benzaldehyde	3.03 × 10^6^
16.201	1,6-Octadien-3-ol, 3,7-dimethyl-	3.80 × 10^6^
16.346	Nonanoic acid, hexyl ester	9.07 × 10^5^
16.685	2-Furancarboxaldehyde, 5-methyl-	5.97 × 10^5^
17.051	3-Cyclohexen-1-ol, 4-methyl-1-(1-methylethyl)-, (R)-	6.28 × 10^5^
17.334	1-Cyclohexene-1-carboxaldehyde, 2,6,6-trimethyl-	1.44 × 10^6^
17.513	2-Dodecenoic acid	3.84 × 10^5^
17.837	2-Furanmethanol	3.15 × 10^5^
17.972	Methyl 4-hydroxybutanoate	7.85 × 10^5^
18.311	L-α-Terpineol	4.16 × 10^6^
18.495	Heptanoic acid, 2-(acetyloxy)-, methyl ester	3.10 × 10^5^
19.036	Oxime-, methoxy-phenyl-_	8.00 × 10^5^
20.140	Heptanoic acid	1.15 × 10^6^
20.285	5,9-Undecadien-2-one, 6,10-dimethyl-, (Z)-	5.00 × 10^5^
20.568	Benzyl alcohol	3.02 × 10^5^
20.766	4-Acetyl-1-methylcyclohexene	1.18 × 10^5^
20.970	Butylated Hydroxytoluene	3.31 × 10^5^
21.315	trans-β-Ionone	8.17 × 10^5^
21.499	1-Dodecanol	2.48 × 10^5^
21.815	Orcinol	9.89 × 10^4^
22.305	1,6,10-Dodecatrien-3-ol, 3,7,11-trimethyl-, [S-(Z)]-	1.76 × 10^5^
22.533	Octanoic acid	2.29 × 10^5^
23.644	Tridecanoic acid	3.83 × 10^5^
24.685	n-Decanoic acid	1.09 × 10^5^
25.712	2(4H)-Benzofuranone, 5,6,7,7a-tetrahydro-4,4,7a-trimethyl-	3.01 × 10^5^
26.432	Benzoic acid	2.70 × 10^5^
26.555	5H-1-Pyrindine	5.57 × 10^5^
26.691	Dodecanoic acid	1.15 × 10^5^
26.980	5-Hydroxymethylfurfural	2.12 × 10^5^
27.813	Decanoic acid, decyl ester	3.59 × 10^5^

**Table A5:** AS sample VOCs.

Retention Time	Name	Mean
2.394	Acetone	4.03 × 10^6^
3.110	Ethyl Acetate	3.14 × 10^6^
4.127	Pentanal	2.52 × 10^6^
6.126	Hexanal	2.12 × 10^6^
6.684	2H-Pyran, 2-ethenyltetrahydro-2,6,6-trimethyl-	1.56 × 10^6^
9.437	3-Dodecyne	7.22 × 10^5^
10.308	3-Tridecene	8.45 × 10^5^
11.327	Acetoin	2.37 × 10^5^
11.708	1-Butanol, 2-methyl-, acetate	5.35 × 10^5^
11.976	Cyclohexanone, 2,2,6-trimethyl-	9.01 × 10^5^
12.200	2-Heptenal, (Z)-	7.19 × 10^5^
12.524	5-Hepten-2-one, 6-methyl-	1.84 × 10^6^
13.633	Nonanal	2.16 × 10^5^
13.755	1H-Pyrazole, 4,5-dihydro-5,5-dimethyl-4-isopropylidene-	1.11 × 10^6^
14.023	1,3-Hexadiene, 3-ethyl-2-methyl-	3.06 × 10^5^
14.142	(1,4-Dimethylpent-2-enyl)benzene	2.37 × 10^5^
14.267	2-Octenal, (E)-	3.15 × 10^5^
14.349	3-Furaldehyde	4.23 × 10^5^
14.469	α-Methyl-α-[4-methyl-3-pentenyl]oxiranemethanol	3.01 × 10^6^
14.671	Ammonium acetate	9.60 × 10^6^
14.932	Furfural	4.37 × 10^7^
15.365	2,4-Heptadienal, (E,E)-	1.60 × 10^5^
15.578	Ethanone, 1-(2-furanyl)-	2.67 × 10^6^
15.876	Benzaldehyde	7.65 × 10^6^
16.143	1,6-Octadien-3-ol, 3,7-dimethyl-	4.83 × 10^6^
16.303	Naphthalene, 1,2,3,4-tetrahydro-1,1,6-trimethyl-	9.30 × 10^5^
16.615	2-Furancarboxaldehyde, 5-methyl-	1.42 × 10^6^
16.840	Furo[3,4-b]furan-2,6(3H,4H)-dione, 4-ethyldihydro-3-methylene-, [3aR-(3aα,4β,6aα)]-	2.39 × 10^5^
16.976	3-Cyclohexen-1-ol, 4-methyl-1-(1-methylethyl)-, (R)-	8.61 × 10^5^
17.276	3-Cyclohexene-1-carboxaldehyde, 1,3,4-trimethyl-	1.85 × 10^6^
17.444	3-Buten-2-one, 3-methyl-4-(1,3,3-trimethyl-7-oxabicyclo[4.1.0]heptan-1-yl)-	7.53 × 10^5^
17.760	2-Furanmethanol	2.59 × 10^5^
17.884	Butanoic acid, 2-methyl-	2.79 × 10^6^
18.151	Bicyclo[3.2.2]non-8-en-6-ol, (1R,5-cis,6-cis)-	7.08 × 10^5^
18.249	L-α-Terpineol	1.08 × 10^7^
18.839	Propanedioic acid, propyl-	5.68 × 10^5^
19.029	Naphthalene, 1,2-dihydro-2,5,8-trimethyl-	5.14 × 10^5^
19.275	Dodecanedioic acid, bis(tert-butyldimethylsilyl) ester	1.76 × 10^5^
19.950	4-Methylphenyl acetone	1.61 × 10^5^
20.067	Heptanoic acid	8.70 × 10^5^
20.228	5,9-Undecadien-2-one, 6,10-dimethyl-, (E)-	5.53 × 10^5^
20.500	Benzyl alcohol	6.87 × 10^5^
20.708	4-Acetyl-1-methylcyclohexene	2.21 × 10^5^
20.926	3,5-Octadiene, 4,5-diethyl-, (E,Z)-	2.10 × 10^5^
21.257	trans-β-Ionone	1.12 × 10^6^
21.466	Cyclopentanol, 1,2-dimethyl-3-(1-methylethenyl)-, [1R-(1α,2β,3α)]-	3.23 × 10^5^
21.746	2,5-Furandicarboxaldehyde	2.24 × 10^5^
21.954	Carbamic acid, N-(3-ethylphenyl)-, phenyl ester	2.96 × 10^5^
22.076	3-Furancarboxylic acid, methyl ester	3.27 × 10^5^
22.247	1,6,10-Dodecatrien-3-ol, 3,7,11-trimethyl-	3.16 × 10^5^
22.455	Octanoic acid	2.15 × 10^5^
23.583	2(3H)-Furanone, 5-hexyldihydro-	3.83 × 10^5^
24.988	Phenol, 2,4-bis(1,1-dimethylethyl)-	2.94 × 10^5^
25.624	2(4H)-Benzofuranone, 5,6,7,7a-tetrahydro-4,4,7a-trimethyl-	4.65 × 10^5^
26.270	Benzoic acid	1.53 × 10^7^
26.889	5-Hydroxymethylfurfural	6.05 × 10^5^
27.803	4a,8a-Butano[1,4]dioxino[2,3-b]-1,4-dioxin, tetrahydro-	1.45 × 10^5^

**Table A6:** SPS sample VOCs.

Retention Time	Name	Mean
1.835	D-Alanine	2.19 × 10^5^
2.118	Dimethyl sulfide	2.55 × 10^5^
4.207	Pentanal	9.61 × 10^6^
5.183	Butanoic acid, ethyl ester	9.08 × 10^5^
5.811	1-Penten-3-one, 2-methyl-	1.53 × 10^6^
5.853	Benzothiophene-3-carboxylic acid, 4,5,6,7-tetrahydro-6-tert-butyl-2-cyclopropanoylamino-, ethyl ester	7.09 × 10^4^
6.121	Hexanal	2.94 × 10^6^
7.200	3-Penten-2-one, (E)-	5.80 × 10^5^
7.664	3-Carene	1.31 × 10^5^
10.180	Hexanoic acid, ethyl ester	1.81 × 10^5^
10.560	1-Pentanol	5.81 × 10^5^
11.401	Acetoin	5.90 × 10^5^
11.449	Octanal	6.62 × 10^5^
11.791	1-Octen-3-one	1.82 × 10^6^
12.261	2-Heptenal, (Z)-	4.58 × 10^6^
12.365	5-Hepten-2-one, 6-methyl-	2.30 × 10^6^
12.685	1-Hexanol	1.76 × 10^5^
13.682	Nonanal	4.64 × 10^5^
13.888	2-Decen-1-ol, (E)-	1.08 × 10^6^
14.020	1,3-Hexadiene, 3-ethyl-2-methyl-	1.20 × 10^5^
14.322	2-Octenal, (E)-	1.47 × 10^6^
14.522	Ethyl 2-(5-methyl-5-vinyltetrahydrofuran-2-yl)propan-2-yl carbonate	6.66 × 10^5^
14.756	Ammonium acetate	6.16 × 10^6^
14.992	Furfural	2.27 × 10^7^
15.303	1-Hexanol, 2-ethyl-	1.46 × 10^5^
15.635	Ethanone, 1-(2-furanyl)-	1.94 × 10^6^
15.929	Benzaldehyde	4.65 × 10^6^
16.197	1,6-Octadien-3-ol, 3,7-dimethyl-	1.50 × 10^7^
16.327	1-Octanol	7.71 × 10^5^
16.929	3(2H)-Furanone, 4-methoxy-2,5-dimethyl-	2.56 × 10^6^
17.095	2,4-Di-tert-butyl-6-(tert-butylamino)phenol	4.56 × 10^4^
17.109	trans-2-Undecen-1-ol	9.08 × 10^4^
17.367	Butanoic acid	3.58 × 10^5^
17.500	Butanoic acid, 4-hydroxy-	7.04 × 10^5^
17.564	2-Decenal, (E)-	4.68 × 10^5^
17.770	Acetophenone	4.31 × 10^6^
17.892	Butanoic acid, 2-methyl-	1.94 × 10^6^
18.130	β-d-Mannofuranoside, phenyl	1.00 × 10^5^
18.138	Octadecanoic acid, 4-methyl-	6.77 × 10^4^
18.305	L-α-Terpineol	4.53 × 10^6^
18.389	6,6,7-Trimethyl-octane-2,5-dione	1.95 × 10^5^
18.415	Hexanoic acid, anhydride	5.08 × 10^5^
18.442	n-Caproic acid vinyl ester	2.52 × 10^5^
18.736	2,6-Octadienal, 3,7-dimethyl-, (E)-	6.19 × 10^5^
18.981	n-Octylsuccinic anhydride	8.99 × 10^5^
19.035	Oxime-, methoxy-phenyl-_	6.87 × 10^5^
19.228	Pseduosarsasapogenin-5,20-dien methyl ether	1.81 × 10^5^
19.276	5α-Cholestan-2-one, oxime	1.86 × 10^5^
19.487	Bicyclo[2.2.1]heptan-2-one, 1-(bromomethyl)-7,7-dimethyl-, (1S)-	3.88 × 10^6^
20.106	Heptanoic acid	2.58 × 10^6^
20.283	5,9-Undecadien-2-one, 6,10-dimethyl-, (Z)-	7.55 × 10^5^
20.559	Benzyl alcohol	3.59 × 10^5^
20.971	Phenylethyl Alcohol	5.90 × 10^5^
21.277	2,3-Dehydro-4-oxo-β-ionol	2.17 × 10^5^
21.322	Stevioside	1.33 × 10^5^
21.486	1-Dodecanol	3.28 × 10^5^
21.627	4,4,8-Trimethyl-non-7-en-2-one	1.60 × 10^5^
21.775	6-Methyl-2-pyrazinylmethanol	4.24 × 10^4^
21.930	Cyclopropanemethanol, α,2-dimethyl-2-(4-methyl-3-pentenyl)-, [1α(R*),2α]-	4.83 × 10^4^
21.955	Phenol	5.52 × 10^4^
21.984	Phosphonic acid, (p-hydroxyphenyl)-	4.25 × 10^4^
22.301	Nerolidyl acetate	1.04 × 10^7^
22.522	Octanoic acid	3.00 × 10^5^
23.194	2-Pentadecanone, 6,10,14-trimethyl-	2.62 × 10^4^
23.378	2-Furanmethanol, tetrahydro-α,α,5-trimethyl-5-(4-methyl-3-cyclohexen-1-yl)-, [2S-[2α,5β(R*)]]-	9.90 × 10^5^
23.578	2(3H)-Furanone, 5-hexyldihydro-	2.78 × 10^6^
23.925	2H-Pyran, 3,6-dihydro-4-methyl-2-(2-methyl-1-propenyl)-	1.68 × 10^5^
24.136	Epiglobulol	3.83 × 10^5^
24.393	Heptadecanal	2.55 × 10^4^
25.829	gamma.-Dodecalactone	4.77 × 10^5^
26.403	Benzoic acid	4.52 × 10^5^
26.663	Dodecanoic acid	1.88 × 10^5^
26.955	5-Hydroxymethylfurfural	1.16 × 10^5^
27.411	1,2-Benzenedicarboxylic acid, bis(2-methylpropyl) ester	1.58 × 10^5^
27.726	Decanoic acid, decyl ester	1.44 × 10^5^
27.778	4a,8a-Butano[1,4]dioxino[2,3-b]-1,4-dioxin, tetrahydro-	7.34 × 10^4^
27.810	di(Butoxyethyl)adipate	9.58 × 10^4^
29.208	2-Propanol, 1-chloro-, phosphate (3:1)	5.56 × 10^4^

**Table A7:** SVL sample VOCs.

Retention Time	Name	Mean
2.060	Dimethyl sulfide	8.70 × 10^5^
4.132	Pentanal	5.78 × 10^6^
5.156	Butanoic acid, ethyl ester	1.98 × 10^6^
6.135	Hexanal	7.04 × 10^5^
7.195	Cyclopropanecarboxylic acid, 2-pentyl ester	5.51 × 10^5^
9.353	1-Butanol, 3-methyl-	3.11 × 10^5^
10.156	Hexanoic acid, ethyl ester	6.29 × 10^5^
10.516	1-Pentanol	4.40 × 10^5^
11.356	Acetoin	9.62 × 10^5^
11.750	1-Octen-3-one	4.22 × 10^5^
12.218	2-Heptenal, (Z)-	8.23 × 10^5^
12.325	2,5-Hexanedione	4.04 × 10^5^
12.553	5-Hepten-2-one, 6-methyl-	6.21 × 10^5^
13.644	Nonanal	3.32 × 10^5^
13.851	2-Hexen-1-ol, (Z)-	7.47 × 10^5^
14.281	2-Octenal, (E)-	3.27 × 10^5^
14.485	Ethyl 2-(5-methyl-5-vinyltetrahydrofuran-2-yl)propan-2-yl carbonate	8.44 × 10^5^
14.708	Ammonium acetate	6.77 × 10^6^
14.952	Furfural	1.07 × 10^7^
15.264	2-Propyl-1-pentanol	1.37 × 10^5^
15.591	Ethanone, 1-(2-furanyl)-	1.08 × 10^6^
15.893	Benzaldehyde	3.64 × 10^6^
16.159	1,6-Octadien-3-ol, 3,7-dimethyl-	9.20 × 10^6^
16.290	1-Octanol	5.37 × 10^5^
16.559	Propanoic acid, 2-methyl-	3.53 × 10^5^
16.887	3(2H)-Furanone, 4-methoxy-2,5-dimethyl-	2.39 × 10^6^
17.239	Butanoic acid, octyl ester	1.73 × 10^5^
17.340	Heptanoic acid	4.50 × 10^5^
17.475	Butanoic acid, 4-hydroxy-	2.84 × 10^5^
17.727	Acetophenone	6.67 × 10^5^
17.914	Butanoic acid, 2-methyl-	2.87 × 10^6^
18.265	L-α-Terpineol	4.02 × 10^6^
18.446	Sulfurous acid, isohexyl hexyl ester	4.11 × 10^5^
18.486	Hexane, 3-bromo-	3.00 × 10^5^
18.694	Isocaryophillene	7.36 × 10^5^
18.996	Dimethylmalonic acid, monochloride, 2-octyl ester	9.54 × 10^5^
19.245	5β,6β-Epoxy-7α-bromocholestan-3β-ol	3.92 × 10^5^
19.450	Bicyclo[2.2.1]heptan-2-one, 1-(bromomethyl)-7,7-dimethyl-, (1S)-	4.92 × 10^6^
20.088	Heptanoic acid	2.85 × 10^6^
20.254	2-Piperidinone, N-[4-bromo-n-butyl]-	5.61 × 10^5^
20.523	Benzyl alcohol	6.02 × 10^5^
20.932	Phenylethyl Alcohol	2.00 × 10^5^
21.312	Heptanoic acid	6.97 × 10^4^
22.118	Humulane-1,6-dien-3-ol	9.67 × 10^4^
22.262	Nerolidyl acetate	1.11 × 10^7^
22.479	Octanoic acid	2.40 × 10^5^
22.852	Cyclohexanone, 3-vinyl-3-methyl-	6.77 × 10^4^
23.325	2-Furanmethanol, tetrahydro-α,α,5-trimethyl-5-(4-methyl-3-cyclohexen-1-yl)-, [2S-[2α,5β(R*)]]-	3.96 × 10^5^
23.534	2(3H)-Furanone, 5-hexyldihydro-	3.14 × 10^6^
23.925	8-Nonene-2,4-diol, 8-methyl-, (R*,S*)-	2.18 × 10^5^
24.179	Epiglobulol	4.32 × 10^5^
25.788	.gamma.-Dodecalactone	3.77 × 10^5^
26.351	Benzoic acid	7.12 × 10^5^
27.416	1,2-Benzenedicarboxylic acid, bis(2-methylpropyl) ester	7.46 × 10^4^

**Table A8:** S100 sample VOCs.

Retention Time	Name	Mean
2.091	Dimethyl sulfide	9.65 × 10^6^
4.130	Pentanal	6.03 × 10^6^
5.149	Butanoic acid, ethyl ester	1.90 × 10^6^
5.740	1-Penten-3-one, 2-methyl-	8.67 × 10^5^
6.109	Hexanal	1.73 × 10^6^
7.168	3-Buten-2-one, 3-methyl-	7.64 × 10^5^
8.992	Cyclobutane, 1,2-bis(1-methylethenyl)-, trans-	1.69 × 10^7^
10.334	gamma.-Terpinene	1.60 × 10^6^
10.500	1-Pentanol	2.75 × 10^5^
10.982	o-Cymene	6.16 × 10^5^
11.343	Acetoin	6.90 × 10^5^
11.455	Octanal	2.79 × 10^5^
11.500	Nonanal	4.33 × 10^5^
11.742	1-Octen-3-one	8.99 × 10^5^
12.210	2-Heptenal, (Z)-	2.60 × 10^6^
12.529	5-Hepten-2-one, 6-methyl-	1.22 × 10^6^
13.807	2-Octene, 2-methyl-6-methylene-	7.11 × 10^5^
14.274	2-Octenal, (E)-	8.26 × 10^5^
14.479	Ethyl 2-(5-methyl-5-vinyltetrahydrofuran-2-yl)propan-2-yl carbonate	1.72 × 10^6^
14.704	Ammonium acetate	7.92 × 10^6^
14.942	Furfural	1.88 × 10^7^
15.259	1-Hexanol, 2-ethyl-	2.08 × 10^6^
15.585	Ethanone, 1-(2-furanyl)-	1.67 × 10^6^
15.890	Benzaldehyde	1.55 × 10^7^
16.153	1,6-Octadien-3-ol, 3,7-dimethyl-	9.33 × 10^6^
16.285	1-Octanol	7.25 × 10^5^
16.420	2H-1,4-Benzodiazepin-2-one, 7-chloro-1,3-dihydro-5-phenyl-1-(trimethylsilyl)-	5.76 × 10^5^
16.708	Bicyclo[2.2.1]heptan-2-ol, 1,3,3-trimethyl-	1.06 × 10^6^
16.883	3(2H)-Furanone, 4-methoxy-2,5-dimethyl-	2.25 × 10^6^
16.980	3-Cyclohexen-1-ol, 4-methyl-1-(1-methylethyl)-, (R)-	8.76 × 10^5^
17.146	2-Decen-1-ol, (E)-	2.09 × 10^5^
17.355	Cyclohexanol, 1-methyl-4-(1-methylethenyl)-	1.17 × 10^6^
17.454	Butanoic acid, 4-hydroxy-	6.96 × 10^5^
17.727	Acetophenone	1.52 × 10^6^
17.906	Butanoic acid, 2-methyl-	3.27 × 10^6^
18.260	L-α-Terpineol	1.15 × 10^7^
18.686	Isocaryophillene	9.64 × 10^5^
18.989	1, 1, 5-Trimethyl-1, 2-dihydronaphthalene	9.34 × 10^5^
19.237	5α-Cholestan-2-one, oxime	3.48 × 10^5^
19.442	Bicyclo[2.2.1]heptan-2-one, 1-(bromomethyl)-7,7-dimethyl-, (1S)-	4.56 × 10^6^
20.084	Heptanoic acid	3.32 × 10^6^
20.243	5,9-Undecadien-2-one, 6,10-dimethyl-, (Z)-	6.78 × 10^5^
20.516	Benzyl alcohol	2.44 × 10^6^
20.928	Phenylethyl Alcohol	4.36 × 10^5^
21.308	Stevioside	1.47 × 10^5^
22.071	α-Cadinol	5.56 × 10^6^
22.261	Nerolidyl acetate	1.33 × 10^6^
22.478	Octanoic acid	2.87 × 10^5^
23.328	2-Furanmethanol, tetrahydro-α,α,5-trimethyl-5-(4-methyl-3-cyclohexen-1-yl)-, [2S-[2α,5β(R*)]]-	5.57 × 10^5^
23.528	2(3H)-Furanone, 5-hexyldihydro-	3.30 × 10^6^
23.930	2-Methyl-7-oxabicyclo[2.2.1]heptane	3.06 × 10^5^
24.140	Propanoic acid, 2-(3-acetoxy-4,4,14-trimethylandrost-8-en-17-yl)-	3.31 × 10^5^
25.779	2(3H)-Furanone, 5-heptyldihydro-	4.20 × 10^5^
26.346	Benzoic acid	7.84 × 10^5^

**Table A9:** SL sample VOCs.

Retention Time	Name	Mean
2.905	Ethyl Acetate	1.46 × 10^6^
5.460	Butanoic acid, 2-methyl-, ethyl ester	3.57 × 10^5^
10.195	Hexanoic acid, ethyl ester	4.05 × 10^5^
11.416	Acetoin	7.36 × 10^5^
11.774	2-Cyclopenten-1-one, 3-(acetyloxy)-	1.40 × 10^5^
14.430	Octanoic acid, ethyl ester	7.50 × 10^4^
14.527	Ethyl 2-(5-methyl-5-vinyltetrahydrofuran-2-yl)propan-2-yl carbonate	7.97 × 10^5^
14.670	1-Octen-3-ol	1.64 × 10^5^
14.771	Ammonium acetate	4.18 × 10^6^
14.999	Furfural	1.23 × 10^7^
15.315	1-Hexanol, 2-ethyl-	5.59 × 10^5^
15.634	Ethanone, 1-(2-furanyl)-	1.48 × 10^6^
15.936	Benzaldehyde	1.24 × 10^7^
16.199	1,6-Octadien-3-ol, 3,7-dimethyl-	2.35 × 10^6^
16.341	1-Octanol	7.98 × 10^5^
16.686	2-Furancarboxaldehyde, 5-methyl-	3.77 × 10^5^
16.933	3(2H)-Furanone, 4-methoxy-2,5-dimethyl-	9.20 × 10^5^
17.115	9-Methyl-10,12-hexadecadien-1-ol acetate	2.87 × 10^5^
17.245	Methoxyacetic acid, 2-pentyl ester	2.67 × 10^5^
17.394	Heptanoic acid	4.50 × 10^5^
17.511	Decanoic acid, ethyl ester	3.77 × 10^5^
17.774	Acetophenone	8.89 × 10^5^
17.967	Butanoic acid, 2-methyl-	1.59 × 10^6^
18.165	Octadecane-1,2-diol, bis(trimethylsilyl) ether	4.53 × 10^5^
18.309	L-α-Terpineol	1.47 × 10^6^
18.495	1,5-Heptadien-4-ol, 3,3,6-trimethyl-	3.54 × 10^5^
18.729	Bicyclo[5.2.0]nonane, 2-methylene-4,8,8-trimethyl-4-vinyl-	2.31 × 10^5^
19.035	Oxime-, methoxy-phenyl-_	7.19 × 10^5^
19.263	5α-Cholestan-2-one, oxime	2.35 × 10^5^
19.484	Bicyclo[2.2.1]heptan-2-one, 1-(bromomethyl)-7,7-dimethyl-, (1S)-	2.41 × 10^6^
20.141	Heptanoic acid	1.24 × 10^6^
20.573	Benzyl alcohol	2.85 × 10^5^
20.975	Phenylethyl Alcohol	2.22 × 10^5^
21.084	2,6-Bis(1,1-dimethylethyl)-4-(1-oxopropyl)phenol	7.42 × 10^4^
21.353	Heptanoic acid	2.65 × 10^5^
21.486	Formic acid, decyl ester	2.65 × 10^5^
22.300	Nerolidyl acetate	4.41 × 10^6^
22.531	Octanoic acid	4.04 × 10^5^
23.376	2-Furanmethanol, tetrahydro-α,α,5-trimethyl-5-(4-methyl-3-cyclohexen-1-yl)-, [2S-[2α,5β(R*)]]-	5.82 × 10^5^
23.579	2(3H)-Furanone, 5-hexyldihydro-	7.91 × 10^5^
23.790	Acetohydrazide, 2-(4-morpholyl)-N2-[(4-methylcyclohex-3-enyl)methylene]-	2.98 × 10^5^
24.538	1-(3,3-Dimethyl-but-1-ynyl)-2,2,3,3-tetramethylcyclopropanecarboxylic acid	9.59 × 10^4^
24.682	n-Decanoic acid	1.67 × 10^5^
25.830	2(3H)-Furanone, 5-heptyldihydro-	2.25 × 10^5^
26.429	Benzoic acid	3.39 × 10^5^
26.552	5H-1-Pyrindine	5.89 × 10^5^
26.983	5-Hydroxymethylfurfural	2.03 × 10^5^

**Table A10:** SS sample VOCs.

Retention Time	Name	Mean
1.763	N-Methylallylamine	1.15 × 10^7^
2.395	Acetone	1.28 × 10^7^
2.865	Ethyl Acetate	5.66 × 10^6^
3.335	Ethanol	4.63 × 10^6^
4.055	2,3-Butanedione	1.56 × 10^6^
10.108	Hexanoic acid, ethyl ester	3.14 × 10^5^
10.671	Acetoin	1.40 × 10^6^
11.754	Heptane, 2,3-dimethyl-	2.52 × 10^5^
12.183	1-Heptanol, 2-propyl-	1.28 × 10^5^
13.503	CH3C(O)OCH(CH3)C(O)CH3	2.00 × 10^5^
14.295	2-Octenal, (E)-	5.69 × 10^5^
14.505	Ethyl 2-(5-methyl-5-vinyltetrahydrofuran-2-yl)propan-2-yl carbonate	1.23 × 10^6^
14.649	Ammonium acetate	6.22 × 10^6^
14.823	Furfural	1.35 × 10^7^
15.616	Ethanone, 1-(2-furanyl)-	9.83 × 10^5^
15.795	Benzaldehyde	2.34 × 10^7^
16.123	1,6-Octadien-3-ol, 3,7-dimethyl-	3.87 × 10^6^
16.245	2,3,4-Trifluorobenzoic acid, 4-tetradecyl ester	1.01 × 10^6^
16.848	3(2H)-Furanone, 4-methoxy-2,5-dimethyl-	6.99 × 10^5^
17.343	n-Decanoic acid	4.52 × 10^5^
17.498	Butanoic acid, 4-hydroxy-	2.58 × 10^5^
17.769	Acetophenone	1.21 × 10^6^
17.926	Butanoic acid, 2-methyl-	1.78 × 10^6^
18.264	L-α-Terpineol	2.98 × 10^6^
18.695	Bicyclo[5.2.0]nonane, 2-methylene-4,8,8-trimethyl-4-vinyl-	3.43 × 10^5^
18.968	Oxime-, methoxy-phenyl-_	2.32 × 10^6^
19.457	Bicyclo[2.2.1]heptan-2-one, 1-(bromomethyl)-7,7-dimethyl-, (1S)-	2.02 × 10^6^
20.119	Cyclopentylcarboxylic acid	1.72 × 10^6^
20.529	Benzyl alcohol	5.73 × 10^5^
20.962	Phenylethyl Alcohol	4.96 × 10^5^
21.337	Heptanoic acid	2.65 × 10^5^
21.464	1-Dodecanol	4.72 × 10^5^
21.785	5-Methyl-2-pyrazinylmethanol	2.17 × 10^5^
22.014	Phosphonic acid, (p-hydroxyphenyl)-	2.39 × 10^5^
22.105	2-Butanone, 4-(2,6,6-trimethyl-2-cyclohexen-1-yl)-	5.26 × 10^5^
22.279	Nerolidyl acetate	7.69 × 10^6^
22.506	Octanoic acid	3.98 × 10^5^
23.234	Tetradecanal	1.39 × 10^6^
23.558	2(3H)-Furanone, 5-hexyldihydro-	1.06 × 10^6^
23.769	Formic acid, 2,3-dimethylphenyl ester	2.92 × 10^5^
24.175	Viridiflorol	8.36 × 10^5^
24.627	Decanoic acid, silver(1+) salt	3.79 × 10^5^
25.025	Phenol, 2,6-bis(1,1-dimethylethyl)-	1.99 × 10^5^
26.374	Benzoic acid	4.74 × 10^6^
26.954	5-Hydroxymethylfurfural	4.34 × 10^5^
27.777	Decanoic acid, cyclohexyl ester	3.41 × 10^5^

**Table A11:** CPS sample VOCs.

Retention Time	Name	Mean
1.745	Hydroperoxide, pentyl	1.06 × 10^7^
8.171	β-Myrcene	1.75 × 10^7^
8.266	D-Limonene	8.70 × 10^8^
10.300	Benzenemethanimine	1.26 × 10^6^
10.985	Benzene, 1-methyl-3-(1-methylethyl)-	9.98 × 10^5^
11.270	(+)-4-Carene	2.73 × 10^6^
13.630	Nonanal	1.18 × 10^6^
14.387	Octanoic acid, ethyl ester	2.28 × 10^6^
14.700	Acetic acid	2.76 × 10^6^
14.943	Furfural	5.77 × 10^6^
15.258	2-Propyl-1-pentanol	1.46 × 10^6^
15.324	α-Copaene	1.17 × 10^6^
15.881	Benzaldehyde	1.04 × 10^7^
16.155	1,6-Octadien-3-ol, 3,7-dimethyl-	1.87 × 10^6^
16.297	1-Octanol	1.74 × 10^6^
16.835	Cyclohexane, 1-ethenyl-1-methyl-2,4-bis(1-methylethenyl)-, [1S-(1α,2β,4β)]-	2.08 × 10^5^
16.967	Bicyclo[7.2.0]undec-4-ene, 4,11,11-trimethyl-8-methylene-	4.18 × 10^6^
17.366	Cyclohexanol, 1-methyl-4-(1-methylethenyl)-	3.91 × 10^6^
18.048	p-Menth-8-en-1-ol, stereoisomer	7.96 × 10^5^
18.264	L-α-Terpineol	2.01 × 10^7^
18.679	1.4-Methano-1H-indene, octahydro-4-methyl-8-methylene-7-(1-methylethyl)-, [1S-(1α,3aβ,4α,7α,7aβ)]-	7.58 × 10^5^
19.101	Naphthalene, 1,2,3,5,6,8a-hexahydro-4,7-dimethyl-1-(1-methylethyl)-, (1S-cis)-	1.09 × 10^6^
20.075	5-Hexenoic acid, 5-methyl-	2.54 × 10^5^
20.242	5,9-Undecadien-2-one, 6,10-dimethyl-, (Z)-	1.86 × 10^5^
20.513	Benzyl alcohol	3.75 × 10^6^
20.924	Phenylethyl Alcohol	1.81 × 10^5^
26.307	Benzoic acid	5.70 × 10^5^

**Table A12:** CVL sample VOCs.

Retention Time	Name	Mean
2.394	1H-Tetrazole-1,5-diamine	1.62 × 10^6^
2.530	Acetic acid, 2-[(acetyl)(2,2-dicyanoethenyl)amino]-, ethyl ester	5.84 × 10^4^
3.401	Ethanol	5.19 × 10^5^
9.374	1-Butanol, 3-methyl-	2.60 × 10^5^
11.348	Acetoin	4.80 × 10^5^
13.279	4-Dodecyne	1.55 × 10^5^
13.650	Nonanal	3.49 × 10^5^
13.855	2-Hexen-1-ol, (E)-	3.83 × 10^5^
14.485	Ethyl 2-(5-methyl-5-vinyltetrahydrofuran-2-yl)propan-2-yl carbonate	2.43 × 10^5^
14.718	Ammonium acetate	2.03 × 10^6^
14.949	Furfural	9.86 × 10^6^
15.265	1-Hexanol, 2-ethyl-	1.65 × 10^5^
15.590	Ethanone, 1-(2-furanyl)-	1.03 × 10^6^
15.890	Benzaldehyde	4.14 × 10^6^
16.159	1,6-Octadien-3-ol, 3,7-dimethyl-	2.42 × 10^6^
16.290	1-Octanol	3.22 × 10^5^
16.632	2-Furancarboxaldehyde, 5-methyl-	3.47 × 10^5^
16.855	1-Propene, 3-chloro-2-(chloromethyl)-	1.00 × 10^5^
16.979	3-Cyclohexen-1-ol, 4-methyl-1-(1-methylethyl)-, (R)-	2.04 × 10^5^
17.249	2H-Pyran-2-one, tetrahydro-	4.80 × 10^5^
17.454	Butyrolactone	6.61 × 10^5^
17.913	Butanoic acid, 2-methyl-	3.15 × 10^5^
18.162	1,7-Dimethyl-4-oxa-tricyclo[5.2.1.0(2,6)]decane-3,5,8-trione	2.57 × 10^5^
18.268	L-α-Terpineol	1.07 × 10^6^
18.484	Methyl 7,9-tridecadienyl ether	1.62 × 10^5^
18.989	1, 1, 5-Trimethyl-1, 2-dihydronaphthalene	6.88 × 10^5^
20.090	Heptanoic acid	8.43 × 10^5^
20.262	(1R,2R,3R,5S)-(−)-Isopinocampheol	3.04 × 10^5^
20.519	Benzyl alcohol	3.19 × 10^6^
20.716	4-Acetyl-1-methylcyclohexene	1.44 × 10^5^
20.928	Phenylethyl Alcohol	2.74 × 10^5^
21.292	Acetic acid, 6,6-dimethyl-2-methylene-7-(3-oxobutylidene)oxepan-3-ylmethyl ester	1.43 × 10^5^
22.258	Nerolidyl acetate	2.45 × 10^5^
23.365	Octadecanal	2.70 × 10^5^
23.535	2(3H)-Furanone, 5-hexyldihydro-	3.88 × 10^5^
23.715	Phenol, 2-methoxy-3-(2-propenyl)-	7.59 × 10^4^
25.785	Ethanol, 2-[2-[(tetrahydro-2H-pyran-2-yl)oxy]ethoxy]-	2.81 × 10^4^
25.850	8-Methyl-hexahydro-pyrano[3,2-b]pyran-2-one	7.16 × 10^4^
26.347	Benzoic acid	5.87 × 10^5^
26.645	2-Propenoic acid, 3-(2-hydroxyphenyl)-, (E)-	3.05 × 10^5^
29.615	2-[2-[2-[2-[2-[2-[2-[2-[2-(2-Methoxyethoxy)ethoxy]ethoxy]ethoxy]ethoxy]ethoxy]ethoxy]ethoxy]ethoxy]ethanol	3.46 × 10^4^

**Table A13:** C100 sample VOCs.

Retention Time	Name	Mean
2.375	2-Heptanone, 7,7-dichloro-	7.84 × 10^5^
3.379	Ethanol	2.22 × 10^5^
4.730	11H-Naphtho[1,2-b]thieno[3,4-d]pyran-11-one, 1-amino-3-methyl-	1.47 × 10^6^
8.998	Cyclobutane, 1,2-bis(1-methylethenyl)-, trans-	9.23 × 10^6^
10.336	.gamma.-Terpinene	8.00 × 10^5^
10.986	Benzene, 1,2,4,5-tetramethyl-	1.98 × 10^5^
11.346	Acetoin	5.52 × 10^5^
12.583	2,6-Lutidine 3,5-dichloro-4-dodecylthio-	6.28 × 10^4^
13.268	7-Oxabicyclo[4.1.0]heptane, 1-methyl-4-(1-methylethenyl)-	8.64 × 10^4^
13.525	Acetic acid, 2-ethylhexyl ester	8.29 × 10^3^
13.642	Nonanal	2.11 × 10^5^
13.848	2-Hexen-1-ol, (E)-	3.27 × 10^5^
14.475	Ethyl 2-(5-methyl-5-vinyltetrahydrofuran-2-yl)propan-2-yl carbonate	5.11 × 10^5^
14.712	Ammonium acetate	2.00 × 10^6^
14.945	Furfural	7.46 × 10^6^
15.261	1-Hexanol, 2-ethyl-	1.37 × 10^6^
15.585	Ethanone, 1-(2-furanyl)-	8.11 × 10^5^
15.897	Benzaldehyde	2.52 × 10^6^
16.155	1,6-Octadien-3-ol, 3,7-dimethyl-	1.83 × 10^6^
16.289	1-Octanol	1.94 × 10^5^
16.650	3-Cyclohexen-1-ol, 1-methyl-4-(1-methylethyl)-	1.53 × 10^5^
16.713	Bicyclo[2.2.1]heptan-2-ol, 1,3,3-trimethyl-	5.69 × 10^5^
16.982	3-Cyclohexen-1-ol, 4-methyl-1-(1-methylethyl)-, (R)-	8.19 × 10^5^
17.259	2(3H)-Furanone, dihydro-4-methyl-	2.91 × 10^5^
17.360	p-Menth-8-en-1-ol, stereoisomer	7.02 × 10^4^
17.421	Cyclohexanol, 1-methyl-4-(1-methylethenyl)-	1.00 × 10^6^
17.775	2-Furanmethanol	2.19 × 10^5^
17.910	Butanoic acid, 2-methyl-	5.10 × 10^5^
18.262	L-α-Terpineol	7.82 × 10^6^
18.679	1H-Benzocycloheptene, 2,4a,5,6,7,8,9,9a-octahydro-3,5,5-trimethyl-9-methylene-	3.33 × 10^5^
18.817	Carvone	1.42 × 10^5^
19.010	Naphthalene, 1,2-dihydro-1,5,8-trimethyl-	4.19 × 10^5^
19.152	Naphthalene, 1,2,3,5,6,8a-hexahydro-4,7-dimethyl-1-(1-methylethyl)-, (1S-cis)-	1.20 × 10^5^
20.086	Heptanoic acid	8.43 × 10^5^
20.250	5,9-Undecadien-2-one, 6,10-dimethyl-, (E)-	2.22 × 10^5^
20.720	Nona-3,5-dien-2-one	1.01 × 10^5^
20.931	Phenylethyl Alcohol	4.73 × 10^5^
21.295	Acetic acid, 6,6-dimethyl-2-methylene-7-(3-oxobutylidene)oxepan-3-ylmethyl ester	8.99 × 10^4^
22.255	1,6,10-Dodecatrien-3-ol, 3,7,11-trimethyl-, [S-(Z)]-	2.74 × 10^5^
23.532	2(3H)-Furanone, 5-hexyldihydro-	3.99 × 10^5^
23.711	Phenol, 2-methoxy-3-(2-propenyl)-	1.45 × 10^5^
25.853	.gamma.-Dodecalactone	5.64 × 10^4^
26.354	Benzoic acid	3.24 × 10^5^

**Table A14:** CL sample VOCs.

Retention Time	Name	Mean
2.511	Ethyl Acetate	1.78 × 10^6^
12.525	5-Hepten-2-one, 6-methyl-	1.16 × 10^5^
13.279	7-Oxabicyclo[4.1.0]heptane, 1-methyl-4-(1-methylethenyl)-	8.60 × 10^4^
13.667	Nonanal	1.08 × 10^5^
13.790	1H-Pyrazole, 4,5-dihydro-5,5-dimethyl-4-isopropylidene-	3.13 × 10^5^
14.505	α-Methyl-α-[4-methyl-3-pentenyl]oxiranemethanol	8.22 × 10^4^
14.625	Sulfurous acid, hexyl octyl ester	1.15 × 10^5^
14.779	Ammonium acetate	1.03 × 10^6^
14.990	Furfural	1.51 × 10^7^
15.305	Acetic acid, dichloro-, heptyl ester	7.47 × 10^5^
15.636	Ethanone, 1-(2-furanyl)-	1.83 × 10^6^
15.931	Benzaldehyde	4.84 × 10^7^
16.195	1,6-Octadien-3-ol, 3,7-dimethyl-	1.05 × 10^6^
16.345	2,3,4-Trifluorobenzoic acid, 4-tetradecyl ester	3.43 × 10^5^
16.672	2-Furancarboxaldehyde, 5-methyl-	4.21 × 10^5^
17.273	Ethanol, 2-(2-ethoxyethoxy)-	1.68 × 10^5^
17.327	1-Cyclohexene-1-carboxaldehyde, 2,6,6-trimethyl-	2.80 × 10^5^
17.498	Butanoic acid, 4-hydroxy-	4.08 × 10^5^
17.968	Butanoic acid, 2-methyl-	1.77 × 10^5^
18.305	L-α-Terpineol	1.20 × 10^6^
18.521	Dodecanal	2.45 × 10^5^
18.785	1H-Imidazole, 4,5-dimethyl-	9.47 × 10^4^
18.903	Naphthalene, decahydro-1,4a-dimethyl-7-(1-methylethyl)-, [1S-(1α,4aα,7α,8aβ)]-	6.00 × 10^5^
19.026	1, 1, 5-Trimethyl-1, 2-dihydronaphthalene	1.42 × 10^6^
19.277	N-Acetyl-2-ethylbutan-1-amine	1.38 × 10^4^
20.132	Heptanoic acid	5.47 × 10^5^
20.284	6,8-Nonadien-2-one, 8-methyl-5-(1-methylethyl)-, (E)-	2.48 × 10^5^
20.561	Benzyl alcohol	1.01 × 10^6^
20.755	4-Acetyl-1-methylcyclohexene	1.70 × 10^5^
20.965	Phenol, 2,6-bis(1,1-dimethylethyl)-4-methyl-, methylcarbamate	2.86 × 10^5^
21.085	2,6-Bis(1,1-dimethylethyl)-4-(1-oxopropyl)phenol	1.73 × 10^5^
21.316	2-Butanone, 4-(2,6,6-trimethyl-2-cyclohexen-1-ylidene)-	2.91 × 10^5^
21.477	1-Dodecanol	2.12 × 10^5^
21.809	6-Methyl-2-pyrazinylmethanol	2.53 × 10^5^
22.010	Acetic acid, phenyl ester	1.72 × 10^5^
22.526	Octanoic acid	2.46 × 10^5^
23.244	1H-Indene-4-acetic acid, 6-(1,1-dimethylethyl)-2,3-dihydro-1,1-dimethyl-	1.31 × 10^5^
23.641	Pentadecanoic acid	2.94 × 10^5^
23.757	Eugenol	4.60 × 10^5^
24.541	1-(3,3-Dimethyl-but-1-ynyl)-2,2,3,3-tetramethylcyclopropanecarboxylic acid	2.44 × 10^5^
25.776	Formic acid, 2,4,6-tri-t-butyl-phenyl ester	3.35 × 10^5^
26.430	Benzoic acid	2.09 × 10^5^
26.545	5H-1-Pyrindine	5.30 × 10^5^
26.972	5-Hydroxymethylfurfural	3.01 × 10^5^
28.302	1H-Inden-5-ol, 2,3-dihydro-1,1,3,3-tetramethyl-4,6-bis(1-methylethyl)-	1.20 × 10^5^
28.885	Terephthalic acid, hexyl tridec-2-yn-1-yl ester	2.33 × 10^5^

**Table A15:** CS sample VOCs.

Retention Time	Name	Mean
2.408	Acetone	4.15 × 10^6^
11.348	Acetoin	1.36 × 10^5^
13.265	3-Dodecyne	2.30 × 10^5^
13.635	Nonanal	1.90 × 10^5^
13.751	1H-Pyrazole, 4,5-dihydro-5,5-dimethyl-4-isopropylidene-	3.94 × 10^5^
14.021	1,3-Hexadiene, 3-ethyl-2-methyl-	1.75 × 10^5^
14.473	Ethyl 2-(5-methyl-5-vinyltetrahydrofuran-2-yl)propan-2-yl carbonate	2.16 × 10^5^
14.686	Ammonium acetate	4.18 × 10^6^
14.929	Furfural	1.26 × 10^7^
15.367	2,4-Heptadienal, (E,E)-	3.62 × 10^5^
15.579	Ethanone, 1-(2-furanyl)-	1.52 × 10^6^
15.874	Benzaldehyde	8.93 × 10^7^
16.143	1,6-Octadien-3-ol, 3,7-dimethyl-	1.18 × 10^6^
16.611	2-Furancarboxaldehyde, 5-methyl-	4.04 × 10^5^
16.850	4-Cyclopentene-1,3-dione	5.89 × 10^4^
16.990	3-Cyclohexen-1-ol, 4-methyl-1-(1-methylethyl)-, (R)-	1.11 × 10^5^
17.279	1-Cyclohexene-1-carboxaldehyde, 2,6,6-trimethyl-	3.54 × 10^5^
17.432	Butanoic acid, 4-hydroxy-	4.79 × 10^5^
17.888	Butanoic acid, 2-methyl-	7.36 × 10^5^
18.152	3-Cyclohexene-1-acetaldehyde, α,4-dimethyl-	3.44 × 10^5^
18.251	L-α-Terpineol	1.63 × 10^6^
18.975	1, 1, 5-Trimethyl-1, 2-dihydronaphthalene	9.02 × 10^5^
19.875	2-Buten-1-one, 1-(2,6,6-trimethyl-1,3-cyclohexadien-1-yl)-, (E)-	8.59 × 10^4^
20.069	Heptanoic acid	4.08 × 10^5^
20.230	α-Ionone	2.65 × 10^5^
20.502	Benzyl alcohol	2.75 × 10^6^
20.705	3-Buten-2-ol, 4-(2,6,6-trimethyl-2-cyclohexen-1-yl)-, (3E)-	2.31 × 10^5^
20.916	Phenylethyl Alcohol	1.52 × 10^5^
21.259	trans-β-Ionone	3.18 × 10^5^
21.740	2,5-Furandicarboxaldehyde	1.40 × 10^5^
21.955	Phenol	1.57 × 10^5^
22.080	3-Furancarboxylic acid, methyl ester	2.73 × 10^5^
23.699	Eugenol	2.11 × 10^5^
24.410	Heptadecanal	2.78 × 10^5^
24.993	Phenol, 3,5-bis(1,1-dimethylethyl)-	1.94 × 10^5^
25.405	l-(+)-Ascorbic acid 2,6-dihexadecanoate	1.36 × 10^6^
26.258	Benzoic acid	2.30 × 10^7^
26.890	5-Hydroxymethylfurfural	5.10 × 10^5^
